# Pharmacophore anchor models of flaviviral NS3 proteases lead to drug repurposing for DENV infection

**DOI:** 10.1186/s12859-017-1957-5

**Published:** 2017-12-28

**Authors:** Nikhil Pathak, Mei-Ling Lai, Wen-Yu Chen, Betty-Wu Hsieh, Guann-Yi Yu, Jinn-Moon Yang

**Affiliations:** 1 TIGP-Bioinformatics, Institute of Information Science, Academia Sinica, Taipei, 115 Taiwan; 2 Institute of Structural Biology and Bioinformatics, National Tsing Hua University, Hsinchu, 30013 Taiwan; 3 National Institute of Infectious Diseases and Vaccinology, National Health Research Institutes, Zhunan, 35053 Taiwan; 4 Graduate Institute of Immunology, National Taiwan University- College of Medicine, Taipei, 10617 Taiwan; 5 Institute of Bioinformatics and Systems Biology, National Chiao Tung University, Hsinchu, 30050 Taiwan; 6 Department of Biological Science and Technology, National Chiao Tung University, Hsinchu, 30050 Taiwan

**Keywords:** Flaviviral NS3 proteases, DENV NS3 protease, Pharmacophore anchor models, Core and specific anchors, Integrated anchor-based virtual screening

## Abstract

**Background:**

Viruses of the *flaviviridae* family are responsible for some of the major infectious viral diseases around the world and there is an urgent need for drug development for these diseases. Most of the virtual screening methods in flaviviral drug discovery suffer from a low hit rate, strain-specific efficacy differences, and susceptibility to resistance. It is because they often fail to capture the key pharmacological features of the target active site critical for protein function inhibition. So in our current work, for the flaviviral NS3 protease, we summarized the pharmacophore features at the protease active site as anchors (subsite-moiety interactions).

**Results:**

For each of the four flaviviral NS3 proteases (i.e., HCV, DENV, WNV, and JEV), the anchors were obtained and summarized into ‘Pharmacophore anchor (PA) models’. To capture the conserved pharmacophore anchors across these proteases, were merged the four PA models. We identified five consensus core anchors (CEH1, CH3, CH7, CV1, CV3) in all PA models, represented as the “Core pharmacophore anchor (CPA) model” and also identified specific anchors unique to the PA models. Our PA/CPA models complied with 89 known NS3 protease inhibitors. Furthermore, we proposed an integrated anchor-based screening method using the anchors from our models for discovering inhibitors. This method was applied on the DENV NS3 protease to screen FDA drugs discovering boceprevir, telaprevir and asunaprevir as promising anti-DENV candidates. Experimental testing against DV2-NGC virus by in-vitro plaque assays showed that asunaprevir and telaprevir inhibited viral replication with EC_50_ values of 10.4 μM & 24.5 μM respectively. The structure-anchor-activity relationships (SAAR) showed that our PA/CPA model anchors explained the observed in-vitro activities of the candidates. Also, we observed that the CEH1 anchor engagement was critical for the activities of telaprevir and asunaprevir while the extent of inhibitor anchor occupation guided their efficacies.

**Conclusion:**

These results validate our NS3 protease PA/CPA models, anchors and the integrated anchor-based screening method to be useful in inhibitor discovery and lead optimization, thus accelerating flaviviral drug discovery.

**Electronic supplementary material:**

The online version of this article (10.1186/s12859-017-1957-5) contains supplementary material, which is available to authorized users.

## Background

Viruses of the family *flaviviridae*, such as Hepatitis C virus (HCV), Dengue virus (DENV), West nile virus (WNV), Japanese encephalitis virus (JEV) etc., cause some of the major viral infections around the world. Among these HCV has been well studied with approved FDA drugs and some inhibitor candidates in clinical trials [1, 2]. However, due to emerging resistance, complications of co-infection and liver damage new treatments for HCV are being pursued [3, 4]. On the other hand, DENV causing dengue fever and life-threatening dengue hemorrhagic fever/dengue shock syndrome [5] still lacks specific therapeutics for treatment and remains a prominent health hazard affecting an estimated 390 million people per year worldwide [6]. Other neglected flaviviruses like WNV [7], JEV [8], MVEV [9], YFV [10] and also the recent ZIKA Virus [11–13] pose high risk to turn into a global epidemic anytime. The lack of effective treatment for these infections [14–16] reminds us of the urgent need to develop novel therapeutics for the infections caused by the *flaviviridae* viruses.

Among the flaviviral proteins, the NS3 protease is an attractive and effective target for antiviral drug development [17–20]. During the viral lifecycle in host cell, the NS3 protease carries out the cleaveage the substrate peptide of viral polyprotein by its conserved catalytic triad *His-Ser-Asp* [21, 22] a critical step is viral replication and survival, which makes the NS3 protease a good drug target. Among the *flaviviridae* family, NS3 protease differs in its cofactor usage; for example, in HCV NS4A acts as cofactor whereas NS2B is cofactor in DENV, WNV, and JEV [5]. Except for HCV NS3 protease inhibitors, none of the inhibitors of DENV, WNV and JEV NS3 proteases have been approved yet [23]. This could be due to the lack of comprehensive guidelines for design and discovery of NS3 protease inhibitors, in spite of some studies finding inhibitors [24, 25]. Also, the screening methods used tend to suffer from lower hit rates and are prone to serotypic efficacy differences [26] and resistance mutations [27].

To deal with these challenges, we proposed the use of pharmacophore anchor based strategy (using site-moiety map [28]) for drug design and discovery of the flaviviral NS3 proteases. In this approach, we developed PA/CPA models for four flaviviral NS3 proteases which contained pharmacophore anchors. We identified five core anchors and several specific anchors indicating common and specific features of NS3 protease respectively. Our PA/CPA models complied with the binding mechanisms of reported NS3 protease inhibitors. An integrated anchor-based screening method using our anchors found three candidates out of which two FDA drugs were active against DENV infection. These results show that our anchors are a valuable asset in targeting NS3 proteases as they provide guidelines for design and discovery of broad/specific inhibitors and also inhibitor hit lead optimization.

## Results

### Overview of PA/CPA models of the flaviviral NS3 proteases

**Fig. 1:**
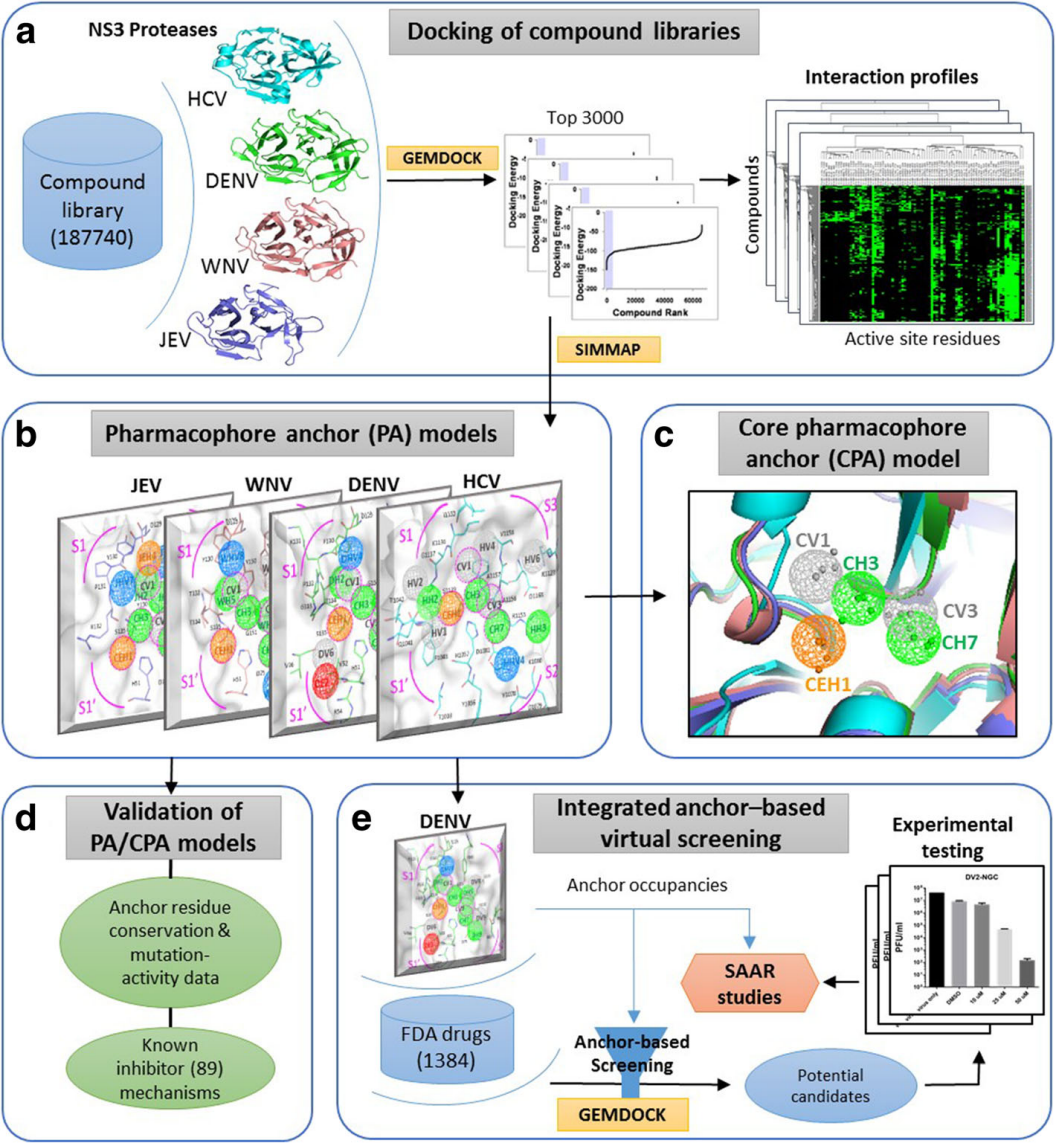
Overview of the PA/CPA models. **a** Docking of the compound library into active sites of HCV, DENV, WNV and JEV NS3 proteases using GEMDOCK. For each of the four proteases, top-ranked 3000 hits (based on best calculated interaction energy) are selected to construct interaction profiles. **b** SimMap analysis of residue-compound interactions leads to Pharmacophore anchor (PA) models of the four NS3 proteases with spatial anchors. **c** Aligning of PA model anchors yields core anchors shown as the Core pharmacophore anchor (CPA) model. **d** Validating the PA/CPA model anchors by anchor residue conservation & mutation-activity data analysis and by understanding known inhibitor mechanisms (**e**) The anchor-based screening carried out for DENV NS3 protease integrates docking by GEMDOCK and the DENV PA model anchors to screen FDA drugs for inhibitor candidates, followed by *invitro* testing and Structure-anchor-activity relationship (SAAR) studies

The overview summarizes our approach in building the PA and CPA models for flaviviral NS3 proteases, elucidating their role in inhibitor binding mechanisms and application in discovering inhibitors (Fig. 1). At first, we docked a 187,740 compound library into the extracted active sites (Methods: Proteins-compound datasets) of four NS3 proteases of HCV, DENV, WNV and JEV (Fig. 1a) using an in-house docking tool GEMDOCK, which has comparable performance to other widely used tools and has been successfully applied to some real world applications [29, 30]. For each protease, the top 3000 compound poses (~0.015%) based on binding energies were selected. Their residue-compound interaction profiles were analyzed for the consensus subsite (residue) –moiety (compound) pharmacophore interactions assigned as anchors using in-house SimMap analysis tool [28]. The anchors with protein active site were represented as pharmacophore anchor (PA) models for each of the four NS3 proteases (Fig. 1b). Next, we aligned these four PA models to find conserved ‘core anchors’ which along with aligned protease active sites formed the CPA model (Fig. 1c). For validating our PA/CPA models, we examined conservation and mutation-activity for anchor residues and explored the binding mechanisms of 89 known NS3 protease inhibitors (Fig. 1d). Finally, we formulated an integrated anchor-based virtual screening and applied it to DENV NS3 protease for screening FDA drugs (Fig. 1e). The potential candidates were tested *invitro* for anti-dengue activity followed by the structure-anchor-activity relationship (SAAR) studies to understand their activities.

### PA and CPA anchor models

**Fig. 2:**
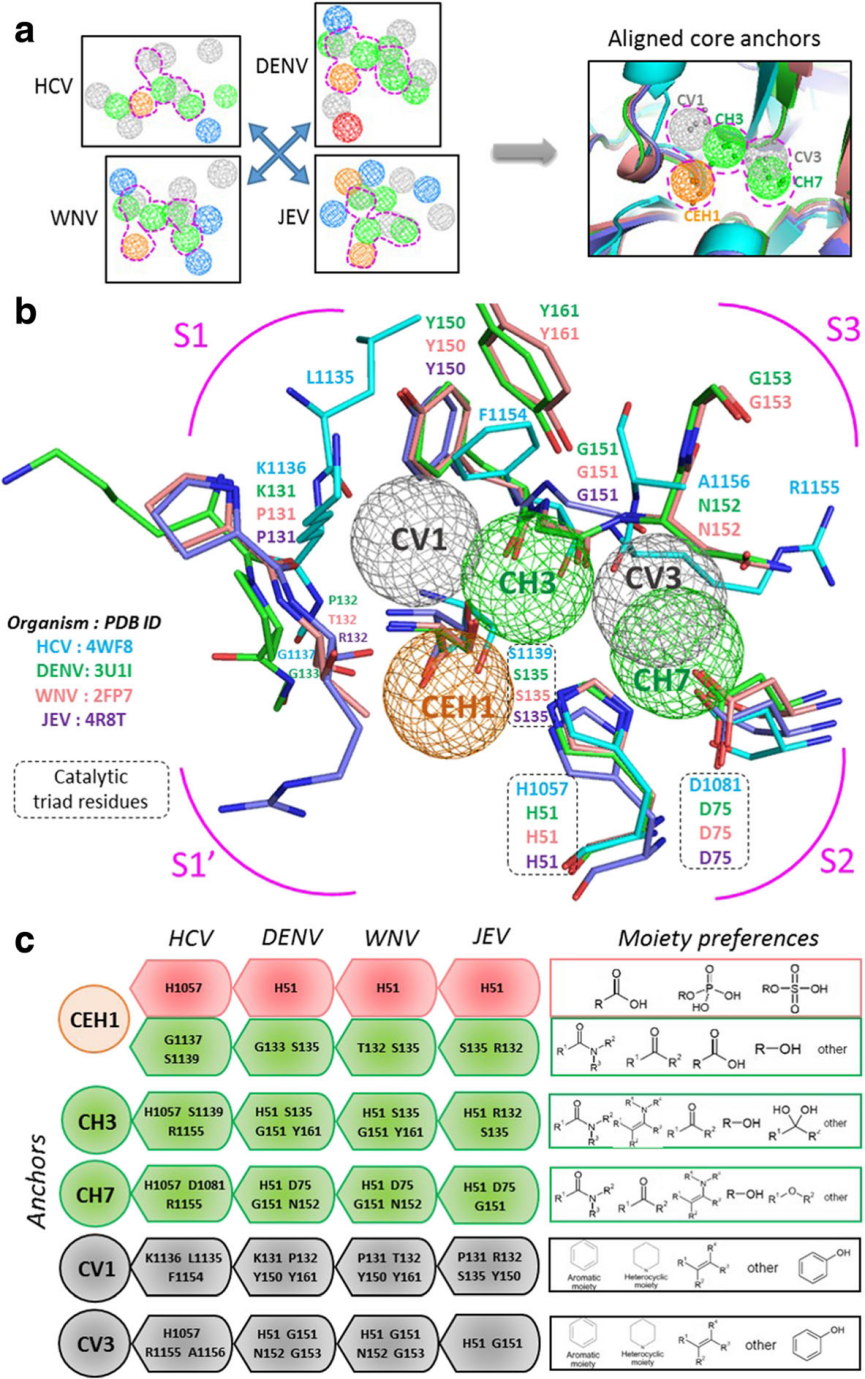
The flaviviral NS3 protease Core pharmacophore anchor (CPA) model. **a** Anchor alignment among the PA models of four virus NS3 proteases (HCV, DENV, WNV and JEV) identified core anchors (pink dotted outline). **b** The CPA model showing five core anchors (CEH1, CH3, CH7, CV1, and CV3) include NS3 proteases from HCV (cyan), DENV (green), WNV (wheat pink), and JEV (purple) with active site subsites (pink). **c** Anchor features of core anchors: anchor types, anchor residues and moiety preferences. (CEH1: orange; CH3 and CH7: green; CV1 and CV3: grey)

The Pharmacophore anchor (PA) model of each NS3 protease depicts their anchors spatially arranged at the active site with features: anchor types (E-H-V), anchor residues and moiety preferences. Additional file 1: Figure S1 summarizes the PA models of the four NS3 proteases (from HCV, DENV, WNV and JEV) in detail. When we aligned these four PA models as in Fig. 2a, we discovered five common core anchors (pink outline) and some specific anchors. The core anchors along with the aligned protease active sites formed the Core Pharmacophore Anchor (CPA) model (Fig. 2b). The flaviviral NS3 protease core and specific anchors (Additional file 1: Figure S2), their involvement in protein function and inhibitory mechanisms are discussed in greater detail in the following sections.

### Core anchors

Core anchors are the conserved subsite (protein residue) - moiety (compound) interactions that often play a critical role in maintaining protein function during the evolution of a protein family. In the current case, they help us understand the conserved interaction features involved in the substrate recognition, proteolysis function and also elucidate inhibitor binding mechanisms across all NS3 proteases of *flaviviridae*. Here, the matching anchors among all aligned NS3 protease PA models were assigned as the core anchors.

Five core anchors were observed in our CPA model including one electrostatic-hydrogen bond anchor (CEH1), two hydrogen bonding anchors (CH3 and CH7), and two van der Waals anchors (CV1 and CV3) (Fig. 2). The CEH1 anchor is located at the oxyanion hole near the subsite S1’ with anchor residues from four viral NS3 proteases: HCV (H1057, G1137, S1139 in blue), DENV (H51, G133, S135 in green), WNV (H51, T32, S135 in red), and JEV (H51, S135, R132 in purple). The CEH1 preferentially interacts with negatively charged moieties (carboxylate, phosphate, and sulfate) and polar groups (carbonyl, ketone) of compounds (Fig. 2c). The CEH1 is involved in substrate stabilization and facilitates catalysis mechanism by its His-Ser catalytic residues (Fig. 2b: in dotted boxes) to cleave the substrate peptide bond [31]. The core CH3 anchor is supported by catalytic Histidine (H1057 in HCV; H51 in DENV, WNV and JEV) and Serine (S1139 in HCV; S135 in DENV, WNV and JEV) along with non-catalytic residues (R1155 in HCV; G151 and Y161 in DENV and WNV; R132 in JEV). The CH3 interacts with the carbonyl groups (Fig. 2c) of the substrate peptide amino acid backbone and stabilizes them during catalysis. The core CH7 anchor occupies the S2-subsite with its residues H1057, D1081, R1155 in HCV; H51, D75, G151, N152 in both DENV and WNV; and H51, D75, G151 in JEV NS3 proteases favoring polar interactions with carbonyl, ketone, amide and alcoholic functional groups. Near the S1 sub-pocket, we observe the hydrophobic CV1 anchor with residues L1135, K1136, and F1154 in HCV; K131, P132, Y150 and Y161 in DENV; P131, T132, Y150, G151, and Y161 in WNV; P131, R132, S135, and Y150 in JEV engaging the substrate P1 side chains by van der Waals interactions. Similarly, CV3 at S2-sub-pocket (with residues H1057, R1155, and A1156 in HCV; residues H51, G151, N152, and G153 in both DENV and WNV; residues H51 and G151 in JEV proteases) offers hydrophobic interactions with its preferred aromatic and heterocyclic moieties.

### Specific anchors

Specific anchors occurring in one or more NS3 proteases often characterize the species-related subtle differences in the binding site sub-pockets and pharmacophore features. Most of the specific anchors appear in more than one PA model, while some of them are unique to only one specific protease. For example, in the four PA models (Additional file 1: Figure S1 and S2), at the S1 sub-pocket the anchors DHV4, WHV8, and JEH4 represent similar pharmacophore environments in DENV, WNV and JEV proteases respectively, but absent in that of HCV. Similarly, specific anchors, DH5-WH2-JH6 and DH2-WH5-JH2, lack a corresponding matched anchor from the HCV PA model. This depicts that the sub-pockets of HCV protease differ distinctly from that of others which is also observed by flaviviral NS3 protease sequence and structure analysis (Additional file 1: Note 2). The Additional file 1: Figure S1 describes the HCV PA model where the anchor HHV4 at S2 subsite matches with that of DH9/DV9 anchors of DENV PA model and WHV4 anchor of WNV PA model, but missing in that of JEV due to the lack of involvement of cofactor residues. Near to the S1’ site of HCV and DENV models, were find the anchors HV1 and DV6 to be matching. Also we observe some unique anchors like HH2, HV2 and HH3 in HCV model, and DE2 anchor exclusive to DENV model (Additional file 1: Figure S1A,B).

These specific anchors often denote the pharmacophore variability at the active site sub-pockets, are critical in assessing protein substrate selectivity-specificity and crucial in selective inhibitor design. For example, in the HCV PA model, the HHV4 anchor at the S2 sub-pocket involves in H-bond and van der Waals interaction without involvement of cofactor residues, while corresponding DH9 H-bond anchor and DV9 van der Waals anchor have similar interactions and anchor moiety preferences mediated by the DENV NS2B cofactor residues G82 and T83. (Additional file 1: Figure S1A,B). This interestingly points out that, in some cases similar pharmacophore interaction environment could be maintained despite of the variable sub-pockets residues among species. We observe that in HCV PA model, the lack of cofactor results in a flat region between S2 and S3 subsites which could be anchored by a unique HH3 anchor. Similarly, at S3 subsite, the HV6 anchor supported by arginine residue in HCV is absent in DENV due to lack of corresponding hydrophobic residues. The unique HH2 and HV2 anchors near the S1’ subsite, help to orient the substrate peptide for proteolytic cleavage. In the DENV PA model, we also find an exclusive DE2 electrostatic anchor supported by R54 offers selectivity for DENV and is missing in the HCV counterpart. Further detailed descriptions of the anchor models can be found in Additional file 1: Note 1.

### Validation of the PA/CPA models

We primarily evaluated our PA/CPA models and anchors by analyzing the evolutionary conservation of anchor residues and effect of their mutation on the protease enzymatic activity (Additional file 1: Note 3, Figure S3 and Table S1). We further verified our models and anchors by applying them to study binding mechanisms and efficacies of 89 known NS3 protease inhibitors of HCV, DENV and WNV NS3 proteases collected (refer to Methods: Proteins-compound datasets). Firstly, the known inhibitors were docked into respective protease active sites, the best binding poses were chosen based on lowest energy and pose similarity to that of bound PDB ligands (from pdb files 4WF8: HCV, 3U1I: DENV, 2FP7: WNV) and then examined the occupation of the PA/CPA model anchors. For a group of inhibitors with variable moieties occupying an anchor, we examined the change in inhibitor activities upon change in moieties at the anchor. This activity differences caused due to variable moiety-anchor interactions at anchor, signify the role of the anchor in inhibitor binding.

We evaluated the HCV PA model using 42 known HCV NS3 protease inhibitors described in Additional file 1: Table S2A. For instance, at the CEH1 anchor (orange circle) occupied by inhibitor R1 groups, the -OH of compound 130 only forms H-bonding, whereas the -NHSO2-() of inhibitor 131 forms both strong electrostatic and H-bond interactions by its ‘SO2’ (charged moiety preferred by CEH1) and ‘NH’ groups, respectively. This leads to an IC_50_ of 75 nM for the active 131 about ~1000 folds more potent than inactive 130. For CH3 anchor, compounds 30, 33 and 1 have similar scaffolds except for –R groups (green circle) which varies from –CH2-, −N(CH3)- to –NH- leading to Ki values 10 μM, 0.12 μM to 0.015 μM respectively (Additional file 1: Table S2A). The change of -R group from aliphatic to polar increases H-bond interactions with the CH3 anchor residues (H1057 and S1139) improving the binding affinities by ~10 fold. Occupying the core CH7 anchor are the varying R1 groups (green circle) of compounds engaging in H-bond interactions. Among R1 groups of compounds 131–134, −O-CO-CH3 group of inhibitor 133 forms stronger H-bond interactions than that of –OH from 131 and –O-CH3 from 132. In the case of the CV1 anchor, the hydrophobic P1 groups of the inhibitors 11–19 fulfill the anchor by VDW interactions. The compounds 18 with long alkyl () P1 group optimally fits at the anchor sub-pocket with Ki value of 13 nM 100-fold better than that of compounds 11 and 13 with shorter functional groups. This is in agreement with the CV1 anchor preference for hydrophobic alkyl chains. Similarly, for the CV3, P2 groups of inhibitors engage the anchor with changing efficacies. The specific HHV4 anchor interacts with the inhibitor -R moieties by both vdW interactions (for compounds 23, 24, 3 and 27) and H-bond interactions (for compounds 48, 60, 53). Similarly, for the specific anchors HH2, HV1, HV2, HV4 and HV6 we observed that variable moiety-anchor interactions by inhibitors guided their activities.

Using the DENV PA model, inhibition mechanisms of 26 known DENV protease inhibitors were explored (Additional file 1: Table S2B). With the core CEH1 anchor, the variable R1 groups (orange circle) of inhibitors 2, 4, 18 and 21 were engaged. When R1 group was electronegative, like –CF3 of compound 18, it was favored by CEH1 by forming strong electrostatic interactions with the anchor residues H51 and S135. Also, −B(OH)2 of compound 21 covalently bonded with catalytic Ser135 of the CEH1 anchor leading to a Kiv value of 0.043 μM. The CH3 anchor is occupied by the inhibitor R3 groups (green circle). The R3 proline group in compound 12, lacks hydrogen on backbone nitrogen to bond with the anchor residues, resulting in binding affinity of 109 μM. Conversely in compound 1 with arginine residue at R1, the main chain –NH- forms H-bonding with anchor thus enhancing efficacy to 5.8 μM. Similar engaging of substrate ligand amino acid backbone by CH3 anchor validates this observation [32]. The side chain of R3 group of inhibitors were observed to interact with the CH7 anchor (Additional file 1: Table S2B). For instance, the inhibitor 7 with arginine side chain forms strong polar bonding at the anchor achieving ~100-fold higher potency compared to inhibitor 6 with threonine side chain. In the same way at CV3, the binding affinity improved as the R3 moiety changed from alanine in 3 (Ki >500 μM) to phenylalanine in 7 (Ki: 40.7 μM) due to increased hydrophobic interactions. Thus the CH7 and CV3 anchors explained the high affinity for arginine(R) at P1 in the substrate K’R’R motif by the DENV NS3 protease. Also in the DENV PA model we found that CV1 and DHV4 anchors favored inhibitor R2 groups (grey circle). The CV1 anchor was better engaged by highly hydrophobic phenylalanine side chain (of inhibitor 6) compared to that of alanine (of inhibitor 2). For inhibitors 10 and 1 at DHV4, arginine side chain of 1 formed stronger H-bonding compared to lysine side chain of inhibitor 10 (also CV3 and DHV4 engage arginine in the substrate).

Twenty one known WNV protease inhibitors, many of them bearing a scaffold similar to Bz-nKKR-H (Additional file 1: Table S2C), were used to explore the WNV PA model. The CEH1 anchor was occupied by R1 -CHO group of compound 25 (IC_50_= 0.271 μM) by bonding with the catalytic anchor residue Ser135. The CH3 anchor was occupied by R3 residue main chain moieties, while CH7 and CV3 anchors were filled by R3 residue side chain atoms. For CH3 anchor, the R1 of compound 7 has alternate amino acid conformation (D-Arg) while that of compound 3 has -N(CH3)-Arg forming weaker H-bond due to methyl substitution. But inhibitor 1 with R1 arginine forms strongest H-bonding interactions with anchor residues and thus the most potent. Similar findings observed at other anchors thus corroborating our WNV PA Model (Additional file 1: Table S2C).

### Integrated anchor-based screening for DENV NS3 protease

To demonstrate the use of PA/CPA models in drug discovery, we proposed an integrated anchor-based virtual screening method which employed pharmacophore anchors from the models in virtual screening to discover true inhibitor hits (Methods: Integrated anchor-based screening approach). Our previous studies showed the applicability of anchors to identify true hit compounds [33, 34]. Here, we employed this strategy against DENV NS3 protease for screening of FDA drug dataset (Methods: Proteins-compound datasets). We obtained three potential FDA drugs as anti-DENV candidates: boceprevir (*Victrelis*) [35], telaprevir (*Incivek*) [36] and asunaprevir (*Sunvepra*) [37]. They originally targeted the HCV NS3 protease and potentially seem to target the homologous DENV NS3 protease following anchors, obtained from PA/CPA models. The binding models and anchor occupancies of these three drug candidates were explored followed by their testing in-vitro for anti-DENV activity.

**Fig. 3:**
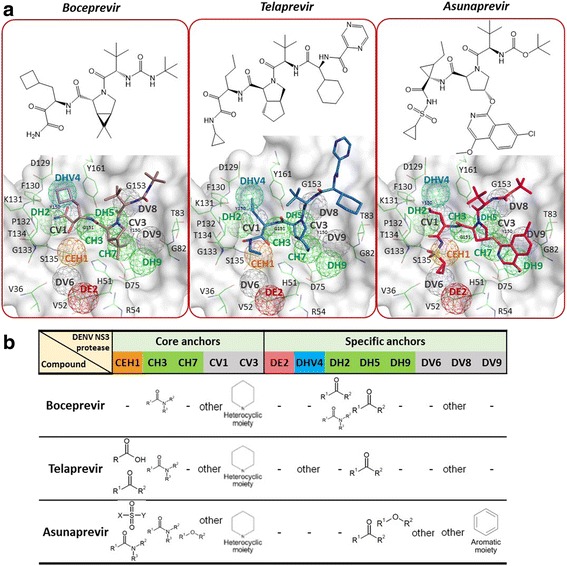
Binding poses and anchor occupancies of inhibitor candidates. **a** Chemical structures of candidates boceprevir, telaprevir and asunaprevir and their binding poses (from docking) in the DENV NS3/2B protease active occupying DENV PA model anchors. **b** Candidate moiety types vs occupied DENV PA model anchors. five core and eight specific anchors are shown colored by their anchor types E (red), H (green), V (grey), E + H (orange), H + V (blue)

For selected candidates boceprevir, telaprevir and asunaprevir the binding poses with best binding energies and anchor occupancies in the DENV PA model were selected (Methods: Integrated anchor-based screening approach) and their binding models were studied (Fig. 3). Boceprevir bound to the DENV NS3 protease occupied three core anchors (CH3, CV1, and CV3) and three specific anchors (DH2, DH5, and DV8) (Fig. 3a). The –CO-NH2- functional group of the boceprevir occupied the CH3 core anchor by H-bonding with residues G151 and Y161, while the pyrrolidine scaffold moiety engaged with the CV3 core anchor by van der Waals interactions (Fig. 3a) and the DH5 anchor was occupied by -N-CO- emerging from pyrrolidone ring, while DV8 anchored the tertiary butyl group. The cyclobutyl group interacted with the CV1 anchor and the adjacent DH2 H-bonded with the terminal –CO-(CO-NH2) functional group of boceprevir. Another candidate, telaprevir occupied CEH1, CH3, CV1, CV3, DHV4, DH5, and DV8 anchors (Fig. 3a). The CEH1 anchor is occupied by –CO-CO-NH-R functional group of telaprevir, by forming electrostatic and H-bond interactions. The –CO-NH2- group fills up the CH3 anchor; pyrrolidine rings interacts with CV3 anchor as in boceprevir; the CV1 and DHV4 anchors were engaged by the propyl group. Final candidate, asunaprevir engaged with all five core anchors and five specific anchors (DH5, DH9, DV6, DV8, and DV9) (Fig. 3a). Its –SO2-NH-R moiety (agreeing with moiety preference for ‘SO2’ moiety; Fig. 3b) engaged in both electrostatic and H-bond interactions with protease core CEH1 anchor residues. While the CH3 anchor engages with carbonyl group which it prefers, CV3 engages pyrrolidine group as in other -previrs. In summary, the candidate compound moieties occupying the anchors are in agreement with the moiety preferences of our PA/CPA models (Fig. 3b).

### Experimental testing of the inhibitor candidates for anti-DENV activity

We tested the potential of the three candidates to inhibit DENV NS3 protease and the viral replication using in-vitro DENV plaque formation assays. Inhibitor candidates were added to the BHK cells infected with DV2-NGC virus strain, and their anti-DENV activity was measured (at various concentrations) by reduction in the viral plaque count (PFU/ml) (Methods: Experimental assays). For this we first determined the highest non-cytotoxic concentrations of the candidates using MTT assay, which was found to be 50 μM (Additional file 1: Figure S4). Then we tested our candidate compounds at various concentrations (going up to 50 μM) for the effect on viral replication by the DENV plaque formation assay. For each concentration, we evaluated the fold change decrease in viral plaque count (PFU/ml) compared to DMSO control. As the amount of viral plaques directly reflected the viral replication, the decrease in viral plaque count (PFU/ml) depicted inhibitory activity.

**Fig. 4:**
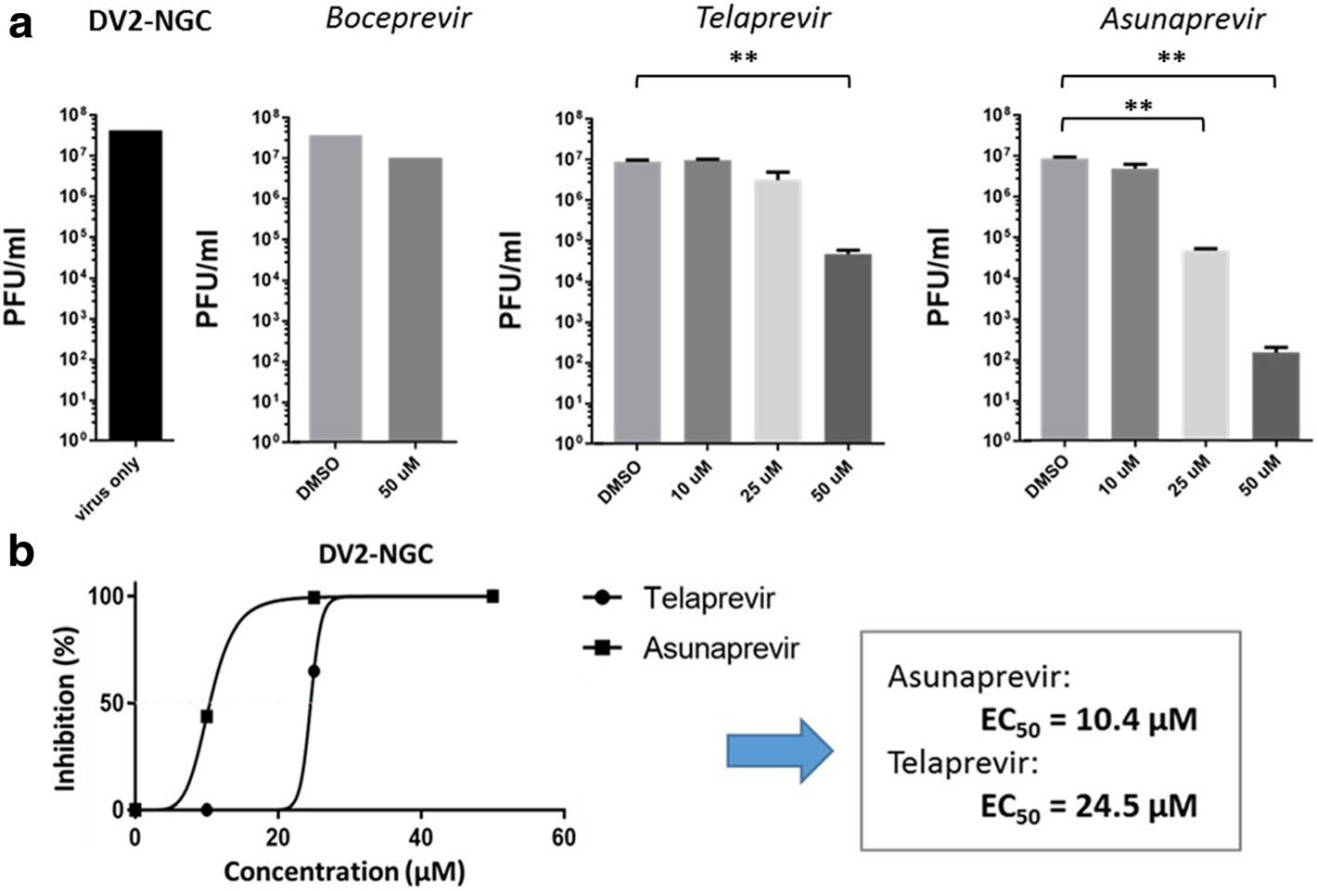
Anti-DENV activities of boceprevir, telaprevir, and asunaprevir by plaque formation assays. Cultured BHK cells were infected with DV2-NGC virus (from Huh7 cell supernatant), treated with DMSO and different concentrations of the inhibitor candidates. After incubation, the viral plaques were quantified and the count (PFU/ml) was recorded. **a** Viral replication is observed by the fold change decrease in plaque count (PFU/ml) on addition of different inhibitor concentrations compared to DMSO control. The statistically significance by one-tailed paired T-test (*n* = 3) is shown [*P < 0.05; **P < 0.01]. **b** Dose vs %inhibition curves were plotted and the EC_50_ values of asunaprevir and telaprevir were observed to be 10.4 μM and 24.5 μM, respectively for DV2-NGC

From the assay results, we observed that the boceprevir did not show any notable decrease in the viral plaques (PFU/ml) in treated cells compared to control at the high concentration of 50 μM (Fig. 4a). However, telaprevir addition to DENV-infected cells resulted in a significant decrease of the viral plaques (PFU/ml) only at 50 μM (Fig. 4a). For asunaprevir, we observed a prominent and significant decrease in the plaque count for both concentrations of 25 μM and 50 μM as compared to DMSO (Fig. 4a). Thus telaprevir and asunaprevir were concluded to actively inhibit DENV replication observed their depletion of viral plaque formation. We then plotted the dose vs % inhibition curves and calculated the EC_50_ values of the two active compounds. The EC_50_ values of asunaprevir and telaprevir were found to be 10.4 μM and 24.5 μM respectively against the DENV2-NGC strain being tested (Fig. 4b). In summary, boceprevir had no observable anti-DENV activity; while telaprevir and asunaprevir were found to be active in inhibiting the DENV replication, with asunaprevir being most active.

### Structure-anchor-activity relationship (SAAR) studies

**Fig. 5:**
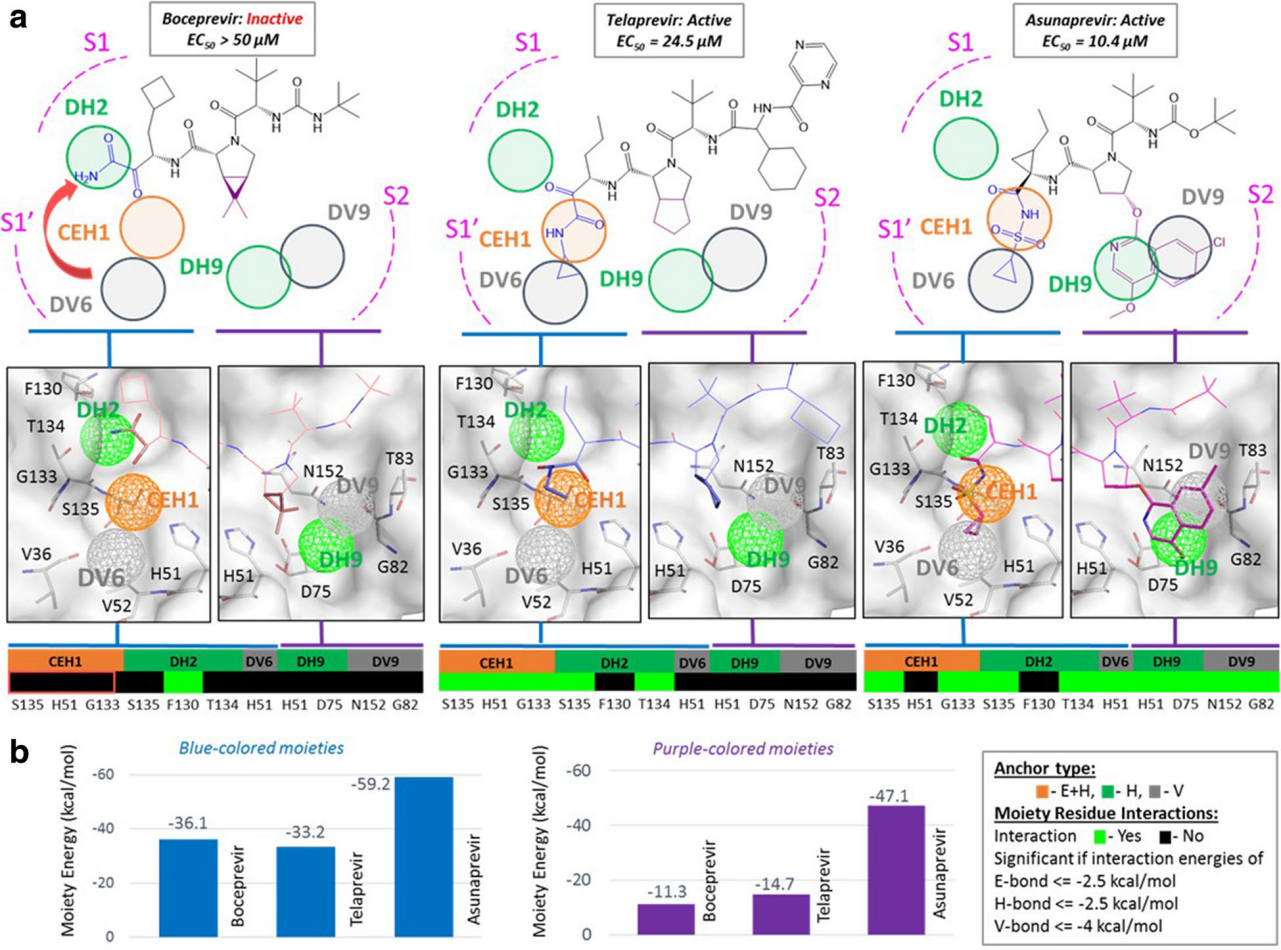
Structure-anchor-activity relationship (SAAR) studies. **a** Boceprevir, telaprevir, and asunaprevir: anti-DENV activities, anchor occupancies and anchor-compound interaction profiles. Corresponding functional groups of the compounds at CEH1, DH2 and DV6 anchors are colored ‘blue’, and near to DH9 and DV9 anchors are colored ‘purple’. The blue moiety of inactive boceprevir (EC_50_ > 50 μM) occupies DH2 anchor but not CEH1 core anchor, while blue moiety of active compounds telaprevir (EC_50_ ~ 20 μM) and asunaprevir (EC_50_ ~ 10 μM) occupy the CEH1 anchor. Further, the purple moiety of asunaprevir occupies two anchors, DH9 and DV9, but not in telaprevir or boceprevir. The protein subsite as shown in the insight, the blue and purple moieties are highlighted as sticks. The anchor-compound interaction profiles show significant E-H-V interactions (bright green). Red rectangle highlights the unoccupancy of CEH1 by boceprevir. **b** Moiety interaction energies of corresponding blue and purple moieties from boceprevir, telaprevir and asunaprevir are depicted

The inhibitor candidates in spite of sharing similar chemical scaffolds and binding poses showed variable anti-DENV activities. To understand this, we pursued Structure-Anchor-Activity Relationship (SAAR) studies by employing our PA/CPA models and anchors to explain binding mechanisms and activities of boceprevir, telaprevir and asunaprevir (Fig. 5). The SAAR studies explored the relationships between compound structures, anchor occupancies and inhibitory activities also revealing determinants and patterns for target inhibition.

On closely examining the candidate binding poses, we found that the compounds showed differential anchor occupancies at S1’ (near the oxyanion hole), S1 and S2 subsites (Fig. 5a). Thus we calculated the interaction energies of compound moieties (blue and purple) with subsite residues. For boceprevir, we noticed that the -CO-CONH2 group (blue-colored) occupied the DH2 anchor at S1 subsite, while CEH1 anchor at S1’ near the oxyanion hole is left empty. The -CO-CONH-R group of telaprevir and the -CONH2-SO2-R group of asunaprevir (both blue-colored) occupied the core CEH1 anchor at S1’ subsite by interacting with its anchor residues H51, S135 and G133. The binding poses of these two drugs (telaprevir and asunaprevir) differ from boceprevir in which flipping (red arrow) of the -CO-CONH2 moiety from S1’ subsite leaves the CEH1 anchor unoccupied, into the S1 subsite to occupy the DH2 anchor. This may be due to lack of an alkyl –R extension on boceprevir -CO-CONH2 moiety to engage DV6 anchor. Conversely, −CO-CONH-R of telaprevir and –CONH-SO2-R of asunaprevir have hydrophobic alkyl –R groups that are stabilized by the DV6 anchor. The interaction profile of inactive boceprevir, confirms CEH1 anchor remaining empty (red outline) and DH2 anchor being occupied. This is further confirmed by Fig. 5b, as the carbonyl group of boceprevir having moiety-interaction energy of −36.1 kcal/mol with DH2 anchor residues, while that of telaprevir and asunaprevir interacted with CEH1 and DV6 anchor residues with −33.2 kcal/mol and −59.2 kcal/mol, respectively. In summary, we learnt that the unoccupancy of CEH1 led to inactivity of boceprevir, while its occupancy facilitated activity of telaprevir and asunaprevir. This observation could be further employed to refine true hits, by prioritizing the compounds occupying the CEH1 anchor.

While examining the active inhibitors telaprevir and asunaprevir, we observed that, the aromatic ring moieties of asunaprevir (purple-colored) extended from the central pyrrolidine scaffold and occupied the specific DH9 and DV9 anchors at the S2 subsite, while corresponding moieties of telaprevir (purple-colored) could not reach these anchors (Fig. 5a). The interaction profile confirms the occupation of DH9 and DV9 anchors by asunaprevir but not by telaprevir. The moiety-residue interaction energy of asunaprevir moiety (purple colored) is −47.1 kcal/mol much higher than −14.7 kcal/mol of corresponding telaprevir moiety (Fig. 5b) confirming our observation of increased asunaprevir binding affinity to the protease resulting its higher efficacy with EC_50_ of 10.4 μM. In summary, the SAAR studies using our PA/CPA models reliably explained *invitro* assay results, revealing the core CEH1 anchor targeting by compounds to be a critical determinant for NS3 protease inhibition and also that higher anchor occupation by compounds leading to better inhibition efficacies.

## Discussion

In our current work, we proposed the PA/CPA models for viral NS3 proteases of the *flaviviridae* family describing the pharmacophore anchors (core and specific) across the family to explore binding mechanisms and to guide drug discovery. One of the challenges for targeting the NS3 protease has been its large and shallow active site which is considered difficult to target [18]. Our PA/CPA models are particularly advantageous here as they reveal anchors which are protein-compound interactions useful in design and discovery of inhibitors with good binding affinities. Such inhibitors targeting anchors could be less prone to encounter drug resistance as the anchor residues they utilize for binding are usually conserved. The core/specific anchors from our PA/CPA models also unveil strategies and guidelines for the design/discovery of broad or selective NS3 protease inhibitors as required.

We chose four flaviviral NS3 proteases and constructed their PA and CPA models using a large compound library dataset. The 187,740 compounds in this library contained diverse functional groups covering large chemical space, resulting in anchor models and anchors being complete, unbiased and independent of this dataset. Our PA/CPA models were able to explain activities of most of the 89 known NS3 protease inhibitors selected for study. However, for few inhibitors, occasionally the anchor occupancies could not exactly explain the mechanisms of binding and activity. Consider an example in WNV protease known inhibitors from Additional file 1: Table S2C, the CEH1 anchor could reflect the activities of compounds 16 and 25 also the CH3 anchor described the activity differences of compounds 7, 3 and 1. However for the CH7 anchor, the compound 1 with Arg group is expected to be more potent than compound 28 with Lys according to anchor occupancy of their binding poses, but compound 28 has better potency. Here, the predicted activity according to docking pose-derived anchor occupancy is erroneous most likely due to the incorrect binding poses of these inhibitors predicted by docking.

We pursued drug repurposing for dengue infection using our integrated anchor-based screening for DENV NS3 protease using a FDA drug dataset (an independent test dataset). This repurposing approach was used as it overcomes the drawbacks of traditionally screening where many inhibitors tend to fail in clinical trials. As a result, we identified three potential candidates of which two FDA drugs asunaprevir and telaprevir as anti-DENV inhibitors with EC_50_ values of 10.4 μM and 24.5 μM respectively. These drugs could be directly proceeded to treat DENV infection or could act as lead compounds for further optimization to obtain better potencies in nanomolar range. It must be noted that the potencies of the two drugs against DENV NS3 protease is in μM range, while they have nM affinities for HCV NS3 protease (IC_50_ for telaprevir ~10 nM, asunaprevir ~1 nM). The differential anchor occupancies of asunaprevir and telaprevir in DENV and HCV NS3 protease active sites are the reason for their differential efficacies (as we observed in our SAAR studies, the pattern of occupied anchors directly affected inhibitor efficacies). Additionally, we feel that the 1384 FDA drug dataset was too small to find novel nanomolar inhibitors, thus further screening of various novel compound sets will be undertaken. These results reveal our models and integrated screening method as robust and useful in effective drug discovery for flaviviral NS3 proteases.

Furthermore, the lead optimization of telaprevir and asunaprevir could be achieved by guidance from our anchor models (using anchor moiety preferences). For example, asunaprevir and telaprevir can be modified by addition of positively charged Arg-like side chain to occupy DHV4 anchor by bonding with D129 anchor residue which would greatly enhance their potencies against DENV protease. Also the compounds efficacies will improve by addition of –COO^−^ group to extend to the DE2 anchor suitably interacting with R54 by electrostatic bonds (Fig. 3a).

## Conclusions

To understand the conserved features, structural intricacies and inhibitor binding mechanisms of the flaviviral NS3 protease, we developed PA/CPA models with pharmacophore anchors for four proteases (HCV, DENV, WNV and JEV). From the models, we discovered five conserved core anchors across *flaviviridae* and several specific anchors unique to one or more species. Our PA/CPA models and anchors were validated (by residue conservation, mutation-activity data) and found to be in agreement with the known protease inhibitors. The Integrated anchor-based screening approach considering compound anchor occupancies was employed for finding true inhibitor hits for DENV NS3 protease. Two FDA drugs telaprevir and asunaprevir (out of the selected three) were found to have anti-DENV activity in-vitro. Thus our integrated screening approach, effectively yielded true inhibitor hits affirming the importance of PA/CPA anchors in drug discovery. Furthermore, SAAR studies used anchors and elaborately elucidated the differences in observed activities of inhibitor hits. We learnt that the occupancy of core anchor CEH1, to be a critical determinant in DENV NS3 protease inhibition. In addition, our PA/CPA anchor moiety preferences could guide lead optimization to enhance efficacy of hit compounds leading to novel and potent inhibitors. Also the current repurposing of FDA drugs telaprevir and asunaprevir for DENV infection shows promise to speed up the therapeutic treatment of dengue infected patients. Moreover, the anchor models of WNV and JEV NS3 proteases facilitate targeting and treatment strategies for these neglected flaviviruses. In conclusion, our work lays a platform for inhibitor design/discovery of *flaviviridae* NS3 proteases boosting up the fight against flaviviral infections.

## Methods

### Proteins-compound datasets

To build our PA/CPA models, we acquired four flaviviral NS3 protease crystal structures from the protein data bank (PDB) (HCV- 4WF8 [38], DENV- 3U1I [32], WNV- 2FP7 [39] and JEV- 4R8T [40]) considered to be in the active forms (based on catalytic triad conformations) suitable for drug discovery (thus related virus MVEV-2WV9 was not selected for PA model building due to its non-active conformation), three of them (4WF8, 3U1I, and 2FP7) being ligand-bound. These four structures were aligned using PyMol protein structure alignment tool and the active sites of these structures were extracted by selecting residues within <= 8°A of that of the aligned bound ligands. To build the PA/CPA model we collected a large compound library of 187,740 compounds, composed of non-redundant compounds of maybridge and natural product datasets from ZINCDB [41].

For the known inhibitor dataset, we collected a total of 89 inhibitors from BindingDB [42] with 42 HCV inhibitors (2.24% of 1869 HCV NS3 protease inhibitors), 26 DENV inhibitors (76.47% of 34 DENV NS3 protease inhibitors) and 21 WNV Inhibitors (58.33% of 36 WNV NS3 protease inhibitors). Due to the large number of HCV NS3 protease inhibitors, we have chosen specific subsets of inhibitors helpful in studying each of the HCV PA model anchors. Also many small molecule inhibitors were not considered in the study as their binding mechanisms could not be elucidated due to inconsistency of their docking poses and PA model anchor occupation. The FDA drug dataset used for repurposing for DENV protease consisted a total of 1384 FDA-approved drugs and was procured from ZINCDB [41].

### Building of the PA/CPA models

The 187,740 compounds were docked into the extracted active sites of each NS3 protease using the in-house docking tool GEMDOCK with docking parameters optimized as per virtual screening protocol and the interaction energies were calculated using the GEMDOCK scoring function [29]. The top-ranked 3000 (~0.015%) compounds with the best interaction energies for each protease were chosen to calculate the interaction profiles (interaction energy map of compounds-protein residues). In the profile an interaction for a compound-residue pair is considered significant, if it has a Z-score > = 1.65 and has the interaction energy (a) E < −2.5 kcal/mol, (b) H < −2.5 kcal/mol, (c) V > −4 kcal/mol. These interaction profiles of top 3000 compounds were analyzed using SiMMap tool [28], and the significant residue-moiety interactions were spatially clustered as pharmacophore ‘anchors’ (shown by mesh spheres as in Fig. 2). Each anchor had three essential features: a) anchor type: E, H and V; b) anchor residues and c) moiety preferences. The NS3 protease active site with spatially arranged anchors formed the pharmacophore anchor (PA) model and such PA models were built for the four flaviviral proteases. The four NS3 protease PA models were then merged by aligning the protease active sites using CE align [43]. The anchors from four corresponding PA models were matched and assigned as ‘core anchors’ if:

- They were within the cutoff distance: (E, H, V) = (3.5°A, 3.5°A, 5.0°A);
- They belonged to the same anchor type E, H or V;
- They occurred in all the proteins of a family;
- They had at least two matching anchor residues within a distance cutoff of: (E, H, V) = (4.5°A, 4.5°A, 5.0°A), with at least one atom (N/O, N/O/S, any heavy atom) within the cutoff;
- They had similar moiety preferences, with at least two preferred moieties matching among the top four preferred moieties for each anchor.

The remaining anchors that did not satisfy the matching criteria were assigned to be ‘specific anchors’.

### Integrated anchor-based screening approach

The integrated anchor-based approach is a docking-based virtual screening method in which the docked compounds are screened based on their occupation of target anchor in addition to their calculated interaction energies with target residues. This approach is effective as it prioritizes the compounds with higher anchor occupancies believed to have higher probability to be true hits [33, 34]. Thus, this integrated approach is applicable to any protein with constructed anchors to enhance hit rates.

Here, we applied this integrated approach to the target case of DENV NS3 protease for screening hits from the 1384 FDA drug dataset. The compounds were first docked into DENV NS3 protease active site (using GEMDOCK virtual screening protocol: 10 poses per compound), followed by calculation of interaction energies and occupancy of anchors (from our DENV PA model). We first selected the top 1000 compounds based on best calculated GEMDOCK interaction energies. From them, the top 100 compound poses with highest anchor occupancy scores were chosen in the next step. Finally, the binding poses and anchor occupancy patterns of these top 100 compounds were compared to that of dengue NS3 protease substrate-mimic ligand Bz-Nle-KKR-H from the PDB ID: 3U1I [32]. The top 10 candidates with poses and interaction profiles similar to substrate were selected. These FDA drugs belonged to the pharmacological category ‘-navir’ and ‘-previr’, known HIV and HCV protease inhibitors respectively. We chose three -previr drugs boceprevir, telaprevir and asunaprevir for further analysis as in addition to being the top candidates for dengue NS3 protease, they also helped in understanding of compound binding mechanisms across HCV and DENV NS3 proteases in relation to our PA/CPA models.

The binding models of the three candidates in the DENV NS3 protease were obtained by selecting the docking poses of the –previr compounds with low calculated interaction energies, adequate occupation of DENV PA model anchors and similar conformation and interactions to that of the ligand in 3U1I.

### Experimental assays

In MTT assay for cytotoxicity, Huh7 cells were plated in a 96-well plate and incubated overnight for cell attachment. Later, the cells were treated with different concentrations of candidate compounds and incubated for 24 h. Then 20 μl/well of MTT solution (5 mg/ml) was added and cells were incubated at 37 °C for 4 h to enable the formation of formazan crystals. The mixture was aspirated and Acid propan-2-ol (0.04 M Hcl in isopropanol) was added to each well to dissolve the dark blue crystals. O.D. values were measured at wavelength 570 nm using ELISA reader (BioRad, iMark™ Microplate Absorbance Reader) and the graphs with O.D. value vs compound concentration were plotted.

For dengue virus plaque formation assay, firstly we cultured BHK (Baby Hamster Kidney fibroblast) cells by seeding them in 6-well plates with 1 × 10^6^ cells per well for at least 12–16 h to allow cell attachment. Cell culture supernatants were obtained from DENV2-NGC virus-infected (m.o.i: 0.5) Huh-7 cells with drug treatment and without drug treatment (only DMSO). The collected supernatants were serially diluted (10^−1^, 10^−2^ to 10^−6^ -fold dilution) and were added to BHK cell monolayer. The plates were shaken every 15 min to ensure that the plate does not dry up. The medium from the plate was removed and 3 ml of DMEM containing 2% FBS and 1% methylcellulose (Sigma-Aldrich) was added. Then the plates were incubated at 37 °C, 5% CO2 for 5 days followed by quantification of the plaques. For this BHK cells in the 6-well plates were fixed and stained with rapid Gram stain crystal violet (East Yao Biotechnology Co., Ltd.) for 2 h. The plaque numbers were counted in triplicate and viral titres (PFU/ml) were calculated, graphs showing viral titres at compound concentrations were plotted.

## Additional file

Additional file 1:
**Note 1**: PA models of HCV, DENV, WNV and JEV NS3 proteases; **Note 2**: Flaviviral NS3 protease sequence and structure analysis; **Note 3:** Evolutionary conservation and mutational analysis of anchor residues. **Figure S1:** Individual pharmacophore anchor (PA) models of four flaviviral NS3 proteases; **Figure S2:** Summary of the core and specific anchors; **Figure S3:** Conservation analysis of core and specific anchor residues of PA/CPA models; **Figure S4.** MTT assays for cytotoxicity studies; **Figure S5:** Flaviviral NS3 proteases: sequence and structure comparison. **Table S1:** Anchor residue mutation-activity data analysis; **Table S2:** PA/CPA model anchor analysis by known inhibitors. (PDF 1784 kb)

## Abbreviations

CPA: Core pharmacophore anchor
DENV2-NGC: dengue virus 2 - New Guinea C strain
NS3: Non-structural protein 3
PA: Pharmacophore anchor
SAAR: Structure-anchor-activity-relationships
