# Supplementary material for: Pharmacophore anchor models of flaviviral NS3 proteases lead to drug repurposing for DENV infection
Source: BMC Bioinformatics. 2017 Dec 28;18(Suppl 16):548. doi: 10.1186/s12859-017-1957-5 (PMC5751397; doi:10.1186/s12859-017-1957-5)
Supplement: Additional file 1: — Note 1: PA models of HCV, DENV, WNV and JEV NS3 proteases; Note 2: Flaviviral NS3 protease sequence and structure analysis; Note 3: Evolutionary conservation and mutational analysis of anchor residues. Figure S1: Individual pharmacophore anchor (PA) models of four flaviviral NS3 proteases; Figure S2: Summary of the core and specific anchors; Figure S3: Conservation analysis of core and specific anchor residues of PA/CPA models; Figure S4. MTT assays for cytotoxicity studies; Figure S5: Flaviviral NS3 proteases: sequence and structure comparison. Table S1: Anchor residue mutation-activity data analysis; Table S2: PA/CPA model anchor analysis by known inhibitors. (PDF 1784 kb) [file 12859_2017_1957_MOESM1_ESM.pdf]

Pharmacophore anchor models of flaviviral NS3 proteases lead to drug repurposing for DENV infection

(Additional file 1)

Contents:

Additional text:

Note 1: PA models of HCV, DENV, WNV and JEV NS3 proteases

Note 2: Flaviviral NS3 protease sequence and structure analysis

Note 3: Evolutionary conservation and mutational analysis of anchor residues

Additional figures:

Figure S1: Individual pharmacophore anchor (PA) models of four flaviviral NS3 proteases

Figure S2: Summary of the core and specific anchors

Figure S3: Conservation analysis of core and specific anchor residues of PA/CPA models

Figure S4: MTT assays for cytotoxicity studies

Figure S5: Flaviviral NS3 proteases: sequence and structure comparison

Additional tables:

Table S1: Anchor residue mutation-activity data analysis

Table S2: PA/CPA model anchor analysis by known inhibitors

Additional text:

PA models of HCV, DENV, WNV and JEV NS3 proteases:

The PA model of the HCV NS3 protease contains 12 distinct anchors, five core (highlighted in pink dotted circles) and 7 specific anchors. The five core anchors have been discussed in the main manuscript, so the general features of seven HCV specific anchors are explained below (Fig. S2A). At the S2 subsite, a specific anchor HHV4 favors both H-bonding (by D1081, D1079 and V1078) and hydrophobic interactions (by H1057, D1081 and Y1056) with compound moieties. Between the S2 and S3 sites we find HH3 anchor (D1168, R1155, D1079) preferring polar groups (like -CO-NH-, -CO- and -COO-) for anchoring the flat region. Near the S1' site, adjacent to CEH1, the HH2 anchor with three residues (Q1041, T1042 and G1137) forms H-bonds with carbonyl and ketone moieties from compounds. Also the anchors HV1 (H1057, Q1041, F1043) and HV2 (Q1041, T1042, G1137) represented the consensus hydrophobic interactions of aromatic and heterocyclic moieties of the docked compounds with subsite residues. Anchors HV4 (K1136, I1132, A1157) and HV6 (R1123, A1156, A1157) at S3 site also favor van der Waals interactions as shown in Figure S2A.

The DENV NS3 protease PA model consists of 13 anchors including 8 specific anchors in addition to 5 core anchors (highlighted in pink) (Fig. S2B). A dual-type DHV4 anchor, an electrostatic DE2 anchor, three H-anchors (DH2, DH5, DH9) and three V-anchors (DV6, DV8 and DV9) constituted the specific anchors. Anchors DHV4 and DH2 at the S1 sub-pocket, accommodated the P1 Arg side chain of the substrate as seen in ligand-bound structure [1]. While the DHV4 anchor offers both H-bonds (by D129 and F130 side chains) and van der Waals bonds (by Y161 and P132), the adjacent DH2 anchor is found buried deep inside the S1 subsite favoring carbonyl and ketone groups to interacts with its polar residues S135, T134 etc. At the S1' subsite, we observe an DENV-exclusive DE2 electrostatic anchor formed by consensus electrostatic interactions of positively charged R54 residue with negatively charged compound moieties ($-PO4^-$ or $-COO^-$). This anchor could be exploited during drug design to achieve selectivity towards inhibiting DENV NS3 protease. Adjacently we find a hydrophobic DV6 anchor (H51, V52 and V36) which helps to stabilize substrate during catalysis. Anchors DH9 and DV9 at the S2 subsite as shown in Figure S2B, are supported by the residues H51, D75, N152 and N152, G82, G83 respectively (from both NS3 and NS2B cofactor chains). Lastly, the S3 site contains anchors DH5 (Y161, G151, N152 and G153) and DV8 (N152, G82, and T83) which prefer polar and hydrophobic groups respectively also interact with P3 group of the substrate [1].

The WNV PA model has 7 specific anchors (WHV4, WHV8, WH2, WH5, WH6, WV5 and WV6) as shown in the Figure 2C. In the WNV NS3 protease ligand-bound crystal structure: 2FP7 [2], at S2 sub-pocket the WHV4 anchor (analogous to HHV4 from HCV PA model) supports P2 Arg side chain of peptide-like inhibitor,

in accordance with its moiety preferences. Similarly, at S1 subsite we find a WHV8 anchor (D129, Y130, P131, Y161), which helps in anchorage of the 'Arg' side chain at P1 region of the inhibitor [2]. Adjacent to WHV8 and deeply located is a WH5 anchor (Y130, T134, S135) available for H-bonding (analogous to DH2). The specific anchor, WH2 (Y161, G151, N152, G153) engages the amino acid backbone carbonyl groups of the peptide substrate by H-bonding. The anchor WV5 forms interaction with compound hydrophobic moieties (like with phenyl group of inhibitor in 2FP7) by anchor residues Y151, G153, V154 and I155. The two anchors WH6 and WV6, engage the substrate/inhibitor functional groups at the S3 subsite of the WNV protease.

In the JEV NS3 protease PA model, we observed 13 anchors (5 core and 8 specific) (Fig. S2D). Among the specific anchors, JHV3, JHV4, JHV7 and JEH4 anchors belonged to the dual interaction type; JH2 and JH6 were H-type; JV1 and JV10 were V-type. In the vicinity of the S1 sub-pocket, we find the three mixed-type anchors and a JH2 anchor. The JHV3 and JHV7 anchors supported H-bonding (with polar amide, ketone, alcohol groups) and van der Waals interactions (with aromatic, alkyl and aliphatic moieties). The JEH4 anchor preferred electrostatic bonding of D129 residue with charged groups like –SO2- of compounds, while residues like Y130 helped in forming H-bond with polar carbonyl moieties. The JH2 anchor located deep in the S1 sub-pocket is analogous to DH2 and WH5 anchors sharing similar binding features. The JH6 (near the core anchor CV3) aids to stabilize the main chains of substrate or peptide inhibitor by H-bonding. At the S3 site, there exists the JHV4 anchor (A125, G151, N152 and G153) H-bonding with polar groups (amide, tertiary amine and alcohol moieties) and forming van der Waals interactions with hydrophobic groups (aromatic, alkyl and phenolic moieties). Finally, JV1 at S3 site and JV10 at S2 site, hold the substrate/inhibitor hydrophobic functional groups in position by van der Waals interactions.

Flaviviral NS3 protease sequence and structure analysis:

We analyzed the four flaviviral NS3 protease sequences by CLUSTALW, a multiple sequence alignment (MSA) tool [3] and structures by CEalign, a structure alignment tool [4]. We observed a significant sequence similarity in aligned NS3 protease (Fig. S1A), except for HCV NS3/4A protease which differs distinctly by the use of NS4A as a cofactor unlike others which used the cofactor NS2B. In addition, the phylogenetic evolutionary tree derived from MSA for NS3 and the co-factors showed that the HCV protease is branched farther away from other viruses denoting its distant evolution (Fig. S1B). The structural alignment revealed a conserved chymotrypsin-like fold with aligned catalytic triad residues *His-Ser-Asp* (Fig. S1C). In DENV, WNV and JEV NS3 proteases, the cofactor NS2B extends to the substrate binding site for substrate stabilization making a deeper active site, but the cofactor 4A in HCV NS3 protease does not extend to the substrate binding pockets resulting in a much flat and wider active site. These subtle similarities and differences in proteases are of great importance in inhibitor design and discovery.

Evolutionary conservation and mutational analysis of anchor residues:

To verify our PA/CPA models, we primarily evaluated anchor-residue conservation by ConsurfDB residue conservation scores [5]. The NS3 protease residues were grouped into four categories as core anchor residues, specific anchor residues, binding site residues (active site residues (<8Å) but not anchor residues) and other residues (not in above groups). Based on the residue conservation scores for four proteases (Fig. S4A), we observed that the >60% of core anchor residues and >40% of specific anchor residues had the highest conservation score of 9. This high conservation of core anchor residues confirms their critical role in the protease structure and function. The specific anchor residues were less conserved compared to core anchors as they tend to have subtle differences among species. Moreover, when comparing the anchor conservation scores (an average of conservation scores of anchor residues) the highest scores 8-9 were attained by core (>60%) and specific (>40%) anchors in all the four proteases (Fig. S4B). This shows that core anchors are critically conserved across the protein family during evolution followed by specific anchors, and points out their key role.

The PA/CPA models were further verified by analyzing the effect of the anchor residue mutations on the overall protease enzymatic activity. For this, we collected the mutation-activity effect data of HCV, DENV, and WNV NS3 protease residues from literature (Table. S2). In general, we observed that anchor residue led to abrogation of enzyme activity depicting their functional role pointing out their targeting for function inhibition. In the HCV NS3 protease (Table. S2A), when His1057 (a catalytic residue) involved in four core and two specific anchors was mutated to Ala, the positively charged His side chain was lost resulting in disruption of the protease activity evident by the inactive mutant. Also, another anchor residue D1081 of CH7 and HHV4 anchors when mutated to Gly, lost it negative charged aspartate side chain leaving the mutant enzyme inactive. But when residue R1123 of specific anchor HV6 mutates to threonine the enzyme retains its biological activity similar to wild type, as both Arg and Thr have similar hydrophobic interactions at the anchor. In the DENV NS3 protease (Table. 2B), the CEH1 anchor residue G133 forming the catalytic oxyanion hole, when mutated to Ala can no longer accommodate oxyanion during catalysis resulting in its undetectable protease activity. Similarly, mutations in residues S135, Y150, G151, N152 and G153 all involved in core and specific anchors resulted in mutant enzymes with undetectable or very low activities. We see similar observations in the residue mutations occurring in the WNV NS3 protease (Table. 2C). Catalytic D75 involved in CH7, WHV4 and WV6 anchors if mutated to Ala lost the ability to stabilize substrate, resulting in an enzymatically inactive enzyme. Interactions of WHV4 and WV6 anchor residues D82 and G83 with substrates are disrupted due to their mutations causing enzyme inactivity. From this analysis, we inferred that enzymatic activity is preserved in mutant when the anchor interaction is preserved in spite of the anchor residue change; while activity is lost when anchor interaction is lost due to residue mutation. Hence, the role of anchors (consensus interactions) in enzymatic activity as well as inhibition is justified.

Additional figures:

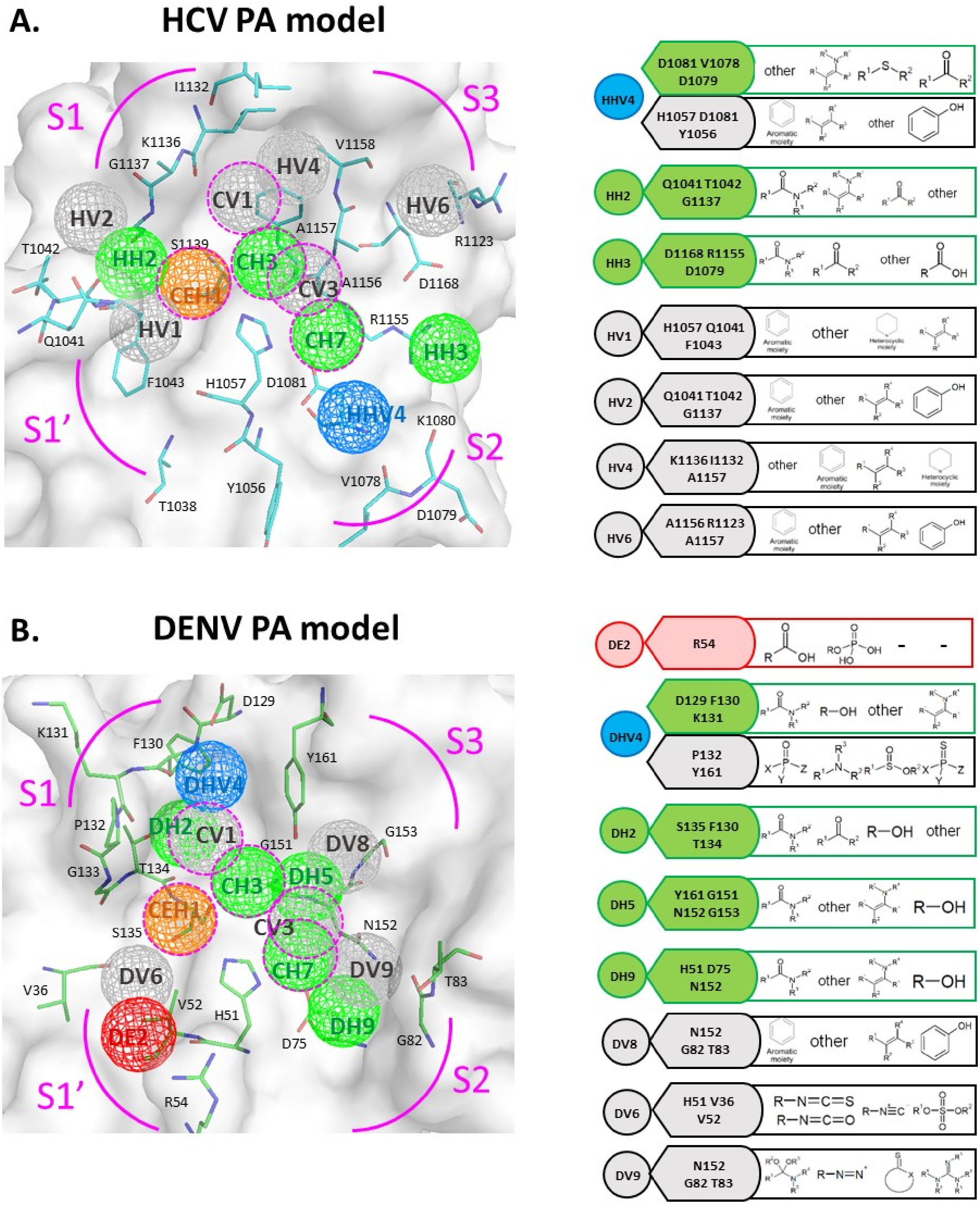

C. WNV PA model

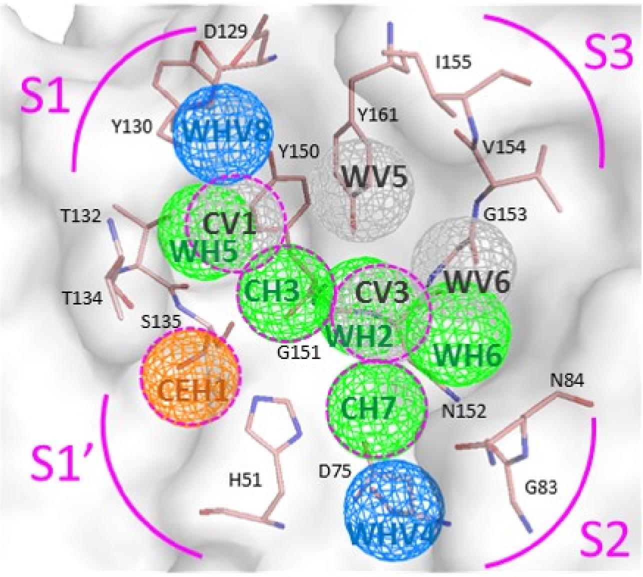

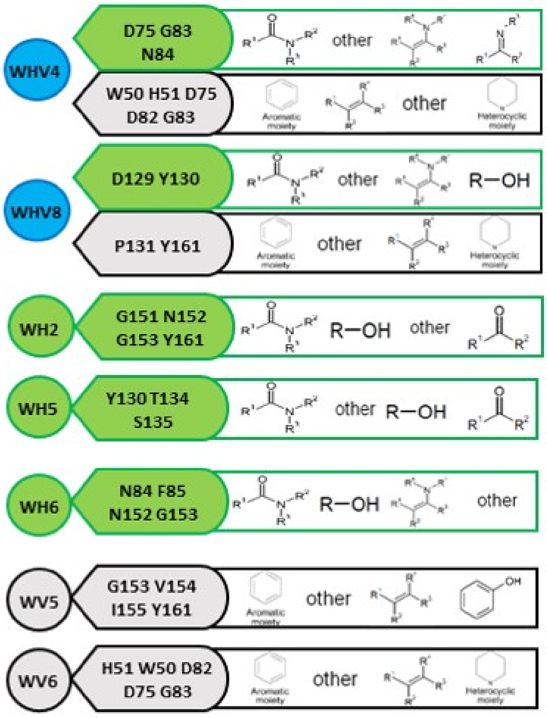

D. JEV PA model

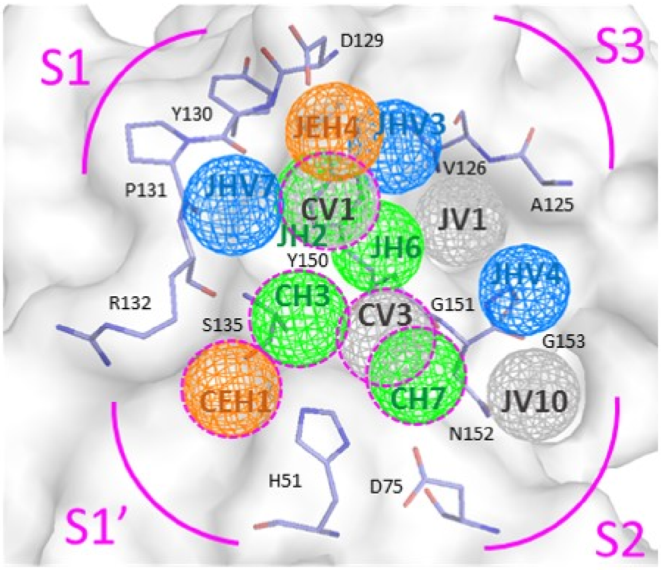

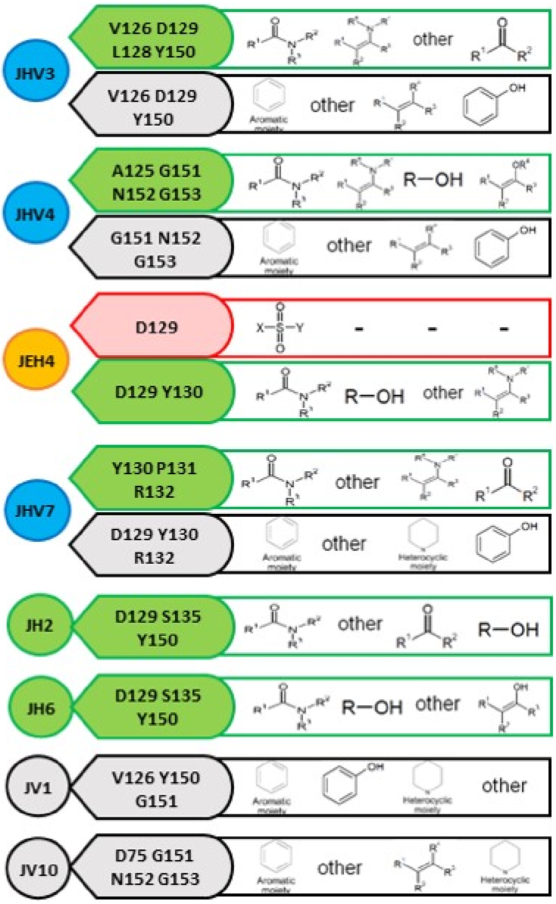

Figure S1. **Individual pharmacophore anchor (PA) models of four flaviviral NS3 proteases:** (A) HCV NS3 protease PA model; (B) DENV NS3 protease PA model; (C) WNV NS3 protease PA model; (D) JEV NS3 protease PA model. Each PA model shows core anchors (dotted pink circles), specific anchors and the protease subsites (pink arcs). The anchor features (type, residues and moiety preferences) for specific anchors are depicted.

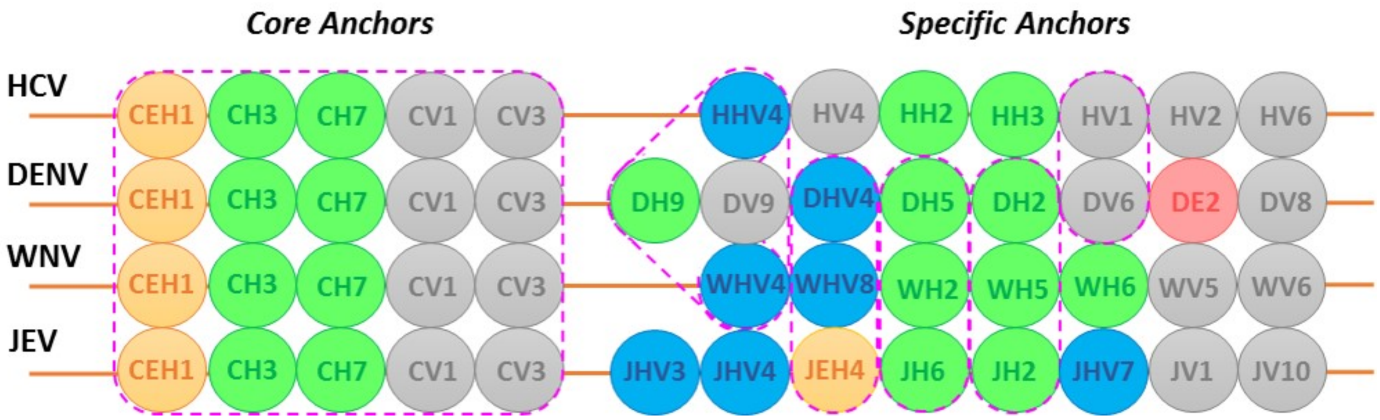

Figure S2. **Summary of the core and specific anchors.** The matched core and specific anchors between proteases are highlighted by dotted pink lines.

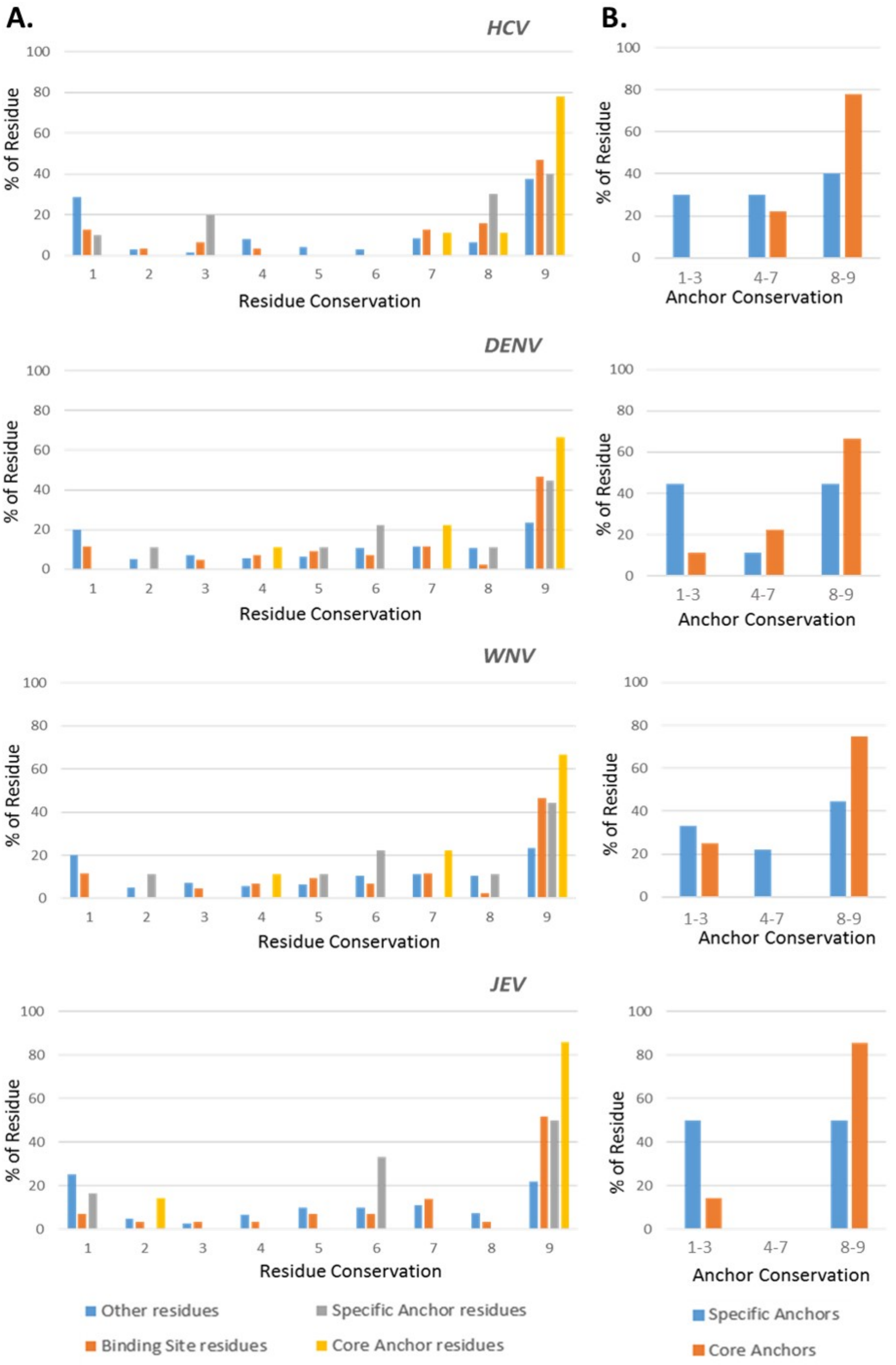
A.
B.
HCV
DENV
WNV
JEV
% of Residue
Residue Conservation
Anchor Conservation
1-3
4-7
8-9
Other residues
Specific Anchor residues
Binding Site residues
Core Anchor residues
Specific Anchors
Core Anchors

Figure S3. **Conservation analysis of core and specific anchor residues of PA/CPA models.** (A) Residue conservation scores and (B) anchor conservation scores. The % of residues with conservation scores (1-9, 1-least conserved, 9-most conserved) for four groups (core anchor residues, specific anchor residues, binding site residues and other residues) in HCV, DENV, WNV and JEV NS3 proteases are shown.

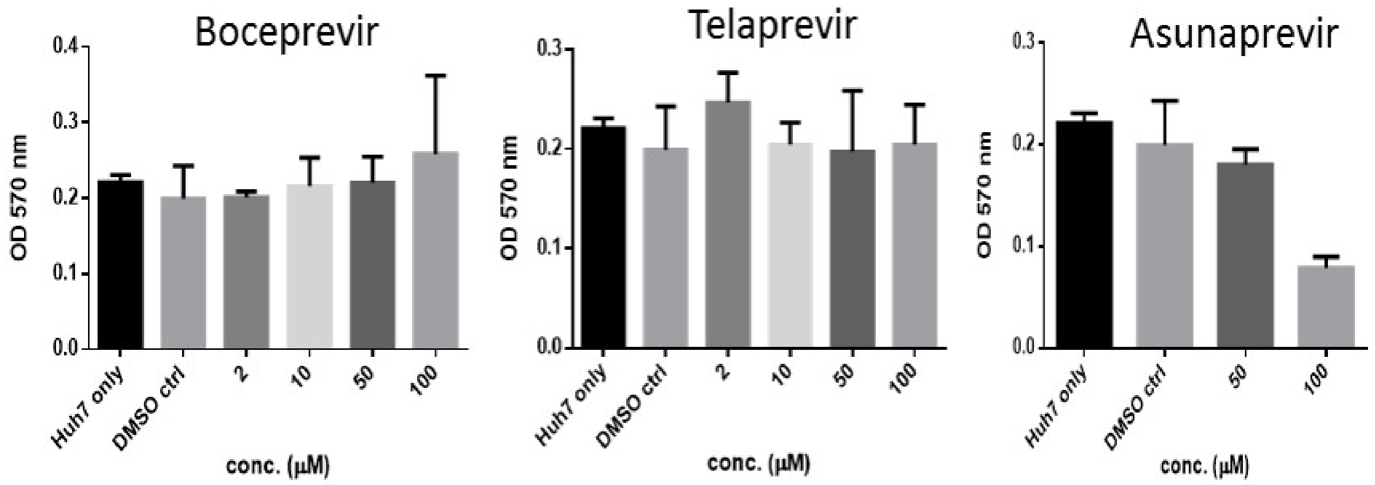

Figure S4. **MTT assays for cytotoxicity studies.** The non-cytotoxic concentrations of the inhibitor candidates are determined by testing increasing compound concentrations (μM) on Huh7 cells, and finding highest concentrations at which majority of cells are viable. OD values at 570 nm represents the cell viability.

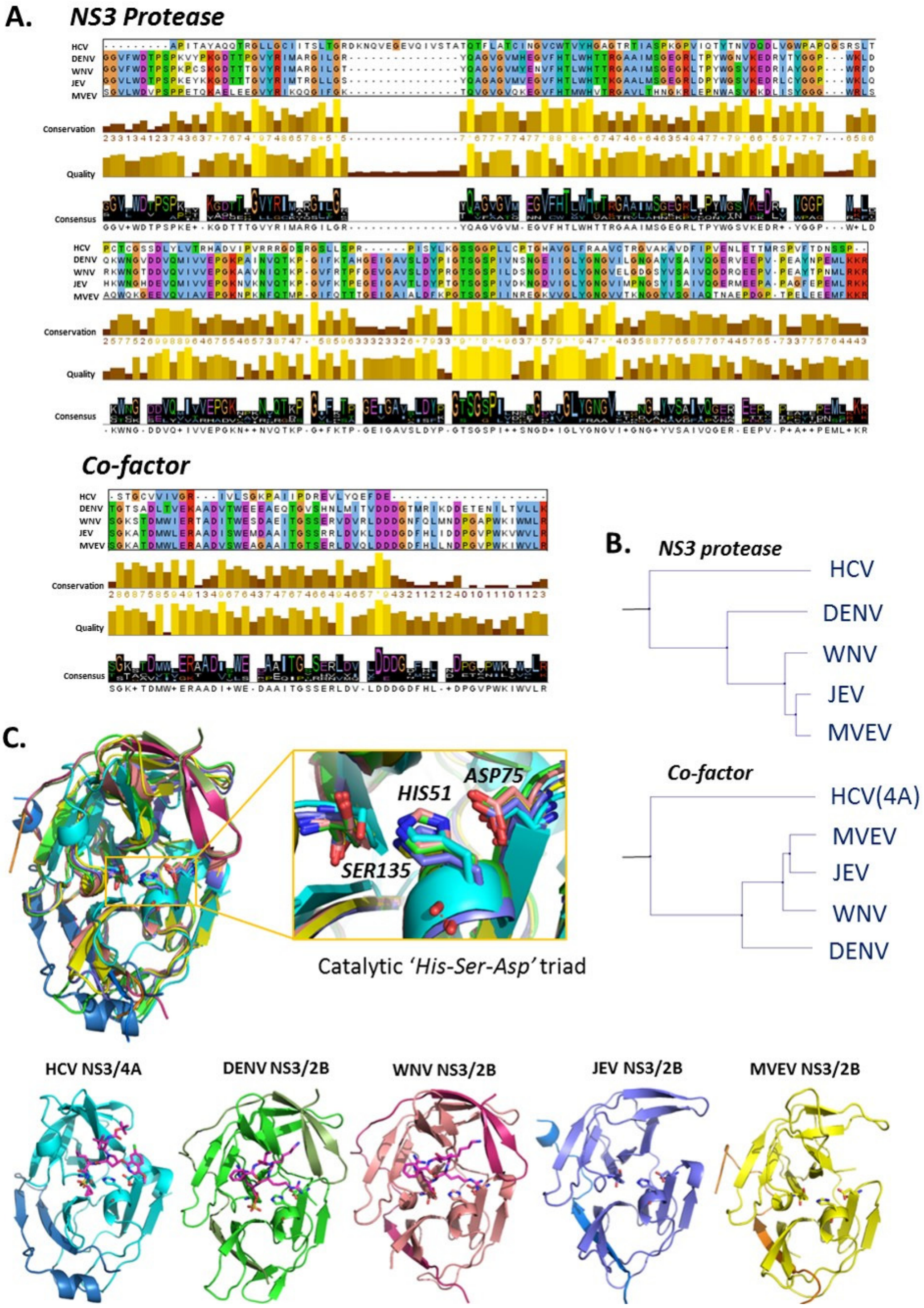

Figure S5. Flaviviral NS3 proteases: sequence and structure comparison. (A) Multiple sequence alignment (MSA) of flaviviral NS3 protease and cofactor (NS4A/NS2B) sequences. (B) Phylogenetic trees based on MSA (by nearest neighborhood method). (C) Aligned flaviviral protease structures with conserved catalytic triad '*His-Ser-Asp*' (in insight). Individual NS3 proteases from HCV (4WF8), DENV (3U1I), WNV (2FP7), JEV (4R8T) and MVEV (2WV9) with conserved protease fold (NS3 and cofactor chains colored differently).

Additional tables:

Table S1. Anchor residue mutation-activity data analysis. The change in residue mutant enzyme activity compared to wild-type (WT) are shown for anchor residues of (A) HCV PA model, (B) DENV PA model and (C) WNV PA model. Mutation types (represented by different fonts): Site directed mutagenesis - normal, Natural variants – italic, Resistance mutations – underlined. Activities (compared to WT): WT-like(WTL): 60%-100%; Decreased(D): 30%-60%; Very less(VL): 5%-30%; Undetectable(UD): 0-5%.

**(A)**<br>**Anchor residue**	**Mutated to**	**Activity (compared to WT)**	**Anchor**	**Reference**
F1043	C<br>S<br>Y	-<br>-<br>-	HV1	[6]<br>[6]<br>[7]
H1057	A	UD	CEH1, CH3, CH7, CV3, HHV4, HV1	[7, 8]
D1079	E<br>H	-<br>-	HHV4, HH3	[7]<br>[7]
D1081	G	UD	CH7, HHV4	[9]
R1123	*T*<br>S	WTL<br>-	HV6	[10]<br>[7]
I1132	N	-	HV4	[7]
L1135	F	-	CV1	[7]
K1136	M<br>R<br>E	D<br>WTL<br>-	CV1, HV4	[11]<br>[11]<br>[7]
S1139	A<br>P	UD<br>UD	CEH1, CH3	[7, 8]<br>[9]
F1154	S<br>G	-<br>-	CV1	[7]
R1155	*Q*<br>T<br>M<br>K<br>S	-<br>-<br>-<br>-<br>D	CH3, CH7, CV3, HH3	[6, 12]<br>[7]<br>[7]<br>[7]<br>[11]
A1156	S<br>T<br>D	-<br>-<br>-	CV3, HV6	[6]<br>[7]<br>[7]

D1168	Q<br>E<br>*V*<br>*Y*<br>*A*<br>*G*	WTL<br>WTL<br>-<br>-<br>-<br>-	HH3	[10]<br>[9]<br>[12, 13]<br>[13]<br>[13]<br>[14]

**(B)<br>Anchor Residue**	**Mutated to**	**Activity (compared to WT)**	**Anchor**	**Reference**
D129	E<br>S<br>A<br>K<br>R<br>L	D<br>D<br>D<br>VL<br>VL<br>VL	DHV4	[15, 16]
F130	Y<br>A<br>S	D<br>VL<br>VL	DHV4, DH2	[15]
K131	S<br>T	WT<br>WT	CV1, DHV4	[15]
G133	A	UD	CEH1	[15, 16]
T134	A<br>D	D<br>UD	DH2	[15, 16]
S135	A<br>C	UD<br>UD	CEH1, CH3, DH2, DH9	[15, 16]
Y150	F<br>A<br>V<br>H	D<br>UD<br>UD<br>UD	CV1, DH9	[15, 16]
G151	A	UD	CH3, CH7, CV3, DH5	[15, 16]
N152	A<br>Q	VL<br>VL	CH7, CV3, DH5, DV8, DV9	[15, 16]
G153	A<br>V	UD<br>UD	CH7, CV3, DH5	[15, 16]

**(C)<br>Anchor Residue**	**Mutated to**	**Activity (compared to WT)**	**Anchor**	**Reference**
D75	A	UD	CH7, WHV4, WV6	[17, 18]

D82	A	VL	WHV4, WV6	[18]
G83	F<br>S<br>D<br>K<br>A	VL<br>VL<br>VL<br>VL<br>UD	WHV4, WV6	[17, 18]
N84	A<br>D<br>E<br>L<br>S	VL<br>WT<br>WT<br>D<br>D	WHV4, WH6	[18, 19]
F85	A	UD	WH6	[18]
D129	A<br>E<br>N	UD<br>VL<br>VL	WHV8	[17]
S135	A	UD	CEH1, CH3, WH5	[17]
Y150	F<br>A	D<br>UD	CV1	[17]
V154	F<br>L	VL<br>D	WV5	[19]
I155	F	D	WV5	[19]

Table S2. PA/CPA model anchor analysis by known inhibitors. (A) HCV protease inhibitors, (B) DENV protease inhibitors and (C) WNV protease inhibitors. For each anchor, there are a group of inhibitors with a common scaffold but with different moieties at the anchor (colored circle). Their activities and efficacies are shown and explained in relation to the anchor.

(A) Anchor	Inhibitor scaffold	Compound	Moiety at anchor	IC_{50}	Ki	Ref.
CEH1	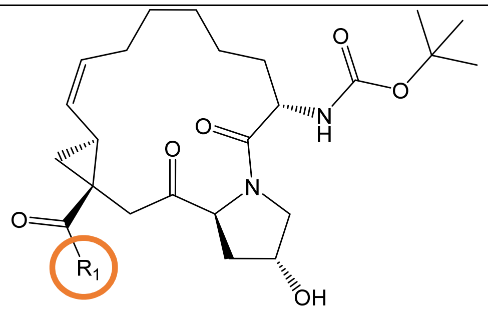	130	-OH	>50000 nM	-	[20]
		131	-NHSO2-(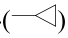)	75 nM		
CH3	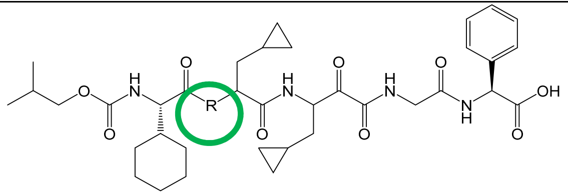	30	-CH2-	-	10 µM	[21]
		33	-N(CH3)-		0.12 µM	
		1	-NH-		0.015 µM	
	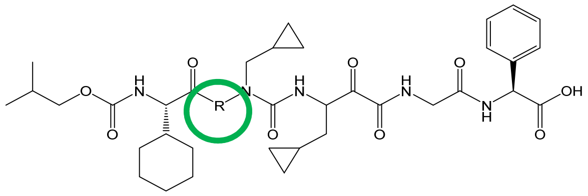	31	-CH2-		2.1 µM	
		32	-N(CH3)-		0.23 µM	
CH7	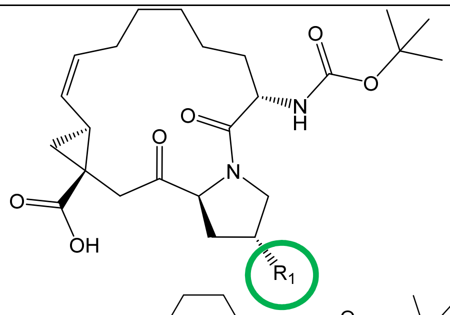	130	-OH	>50000 nM	-	[20]
		4	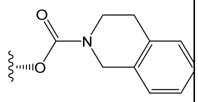	177 nM		
	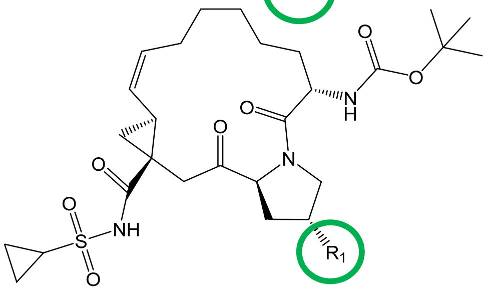	132	-O-CH3	220 nM		
		131	-OH	75 nM		
		133	-O-CO-CH3	12 nM		
		134	-O-C4H9	3 nM		

CV1	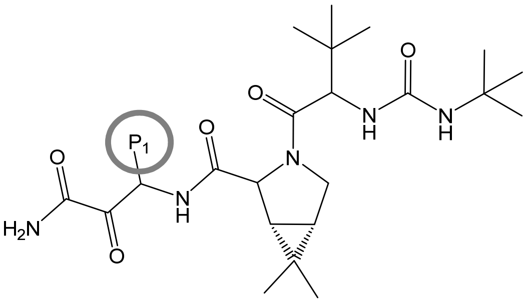	13	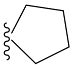	-	1800 nM	[22]
		11	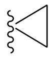		1600 nM	
		12	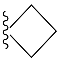		100 nM	
		19	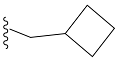		14 nM	
		18	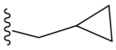		13 nM	
CV3	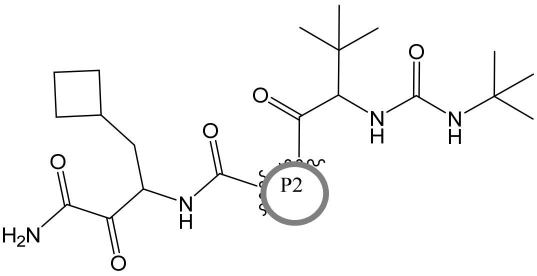	26	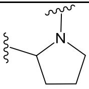	-	5000 nM	[22]
		27b	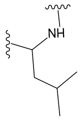		1000 nM	
		37	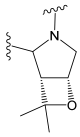		960 nM	
		25	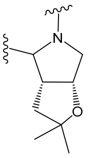		500 nM	
		19	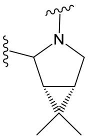		14 nM	
HHV4	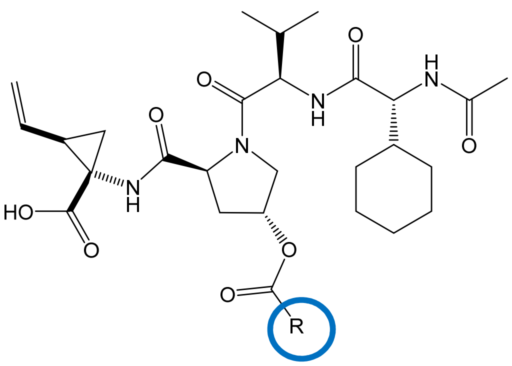	24	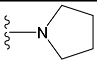	14877 nM	-	[20]
		23	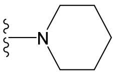	4286 nM		
		3	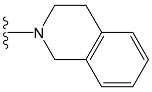	210 nM		
		27	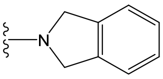	186 nM		
		48	-H	1.2 nM		
		60	-4,7-di-F	0.7 nM		
		53	-4-NH2	0.2 nM		

HH2	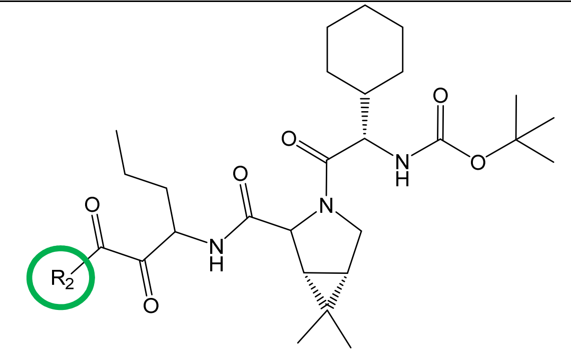	38<br>36<br>37<br>39	-N(CH3)2<br>-NH(CH3)<br>-NH2<br>-OH	-	>13 µM<br>1.5 µM<br>0.1 µM<br>0.11 µM	[23]
HV1	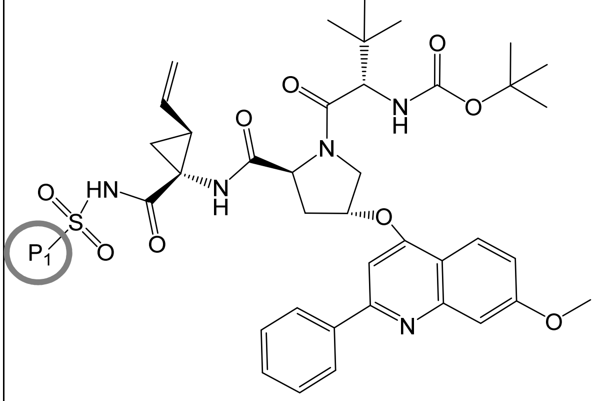	9<br>8<br>7<br>6	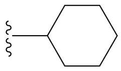<br>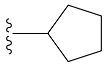<br>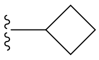<br>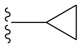	149 nM<br>71 nM<br>7 nM<br>1 nM	-	[24]
HV2	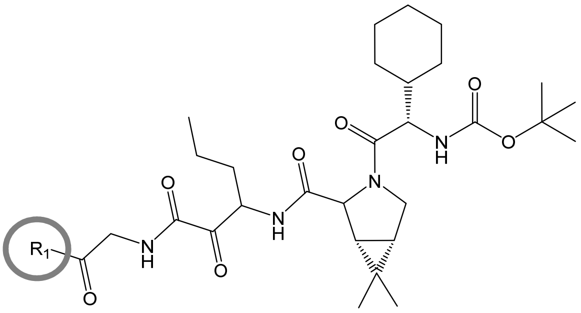	30<br>29<br>26<br>27	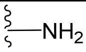<br>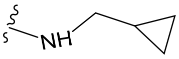<br>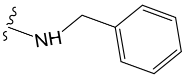<br>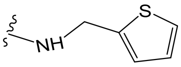	-	0.790 µM<br>0.130 µM<br>0.056 µM<br>0.060 µM	[23]
HV4	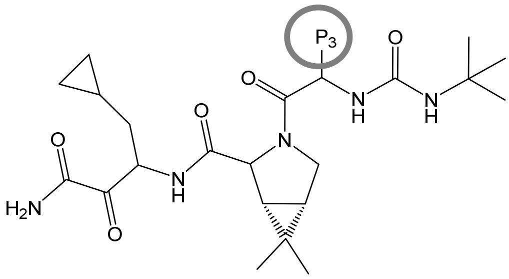	29<br>30<br>18	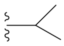<br>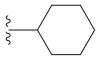<br>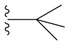	-	100 nM<br>50 nM<br>13 nM	[22]
HV6	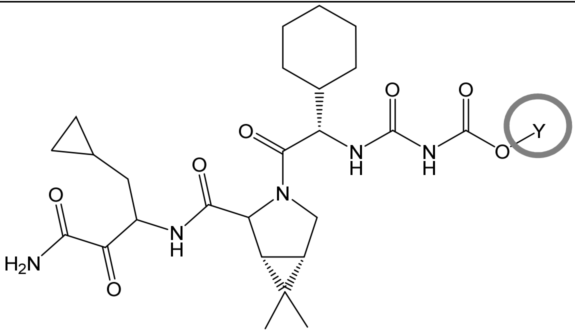	31<br>32<br>33<br>14	-CH3<br>-C2H5<br>-iPro<br>-tBu	-	800 nM<br>230 nM<br>60 nM<br>25 nM	[22]

(B) Anchor	Inhibitor scaffold	Compo-und	Moiety at anchor	% Inhibition	Ki	Ref.
CEH1	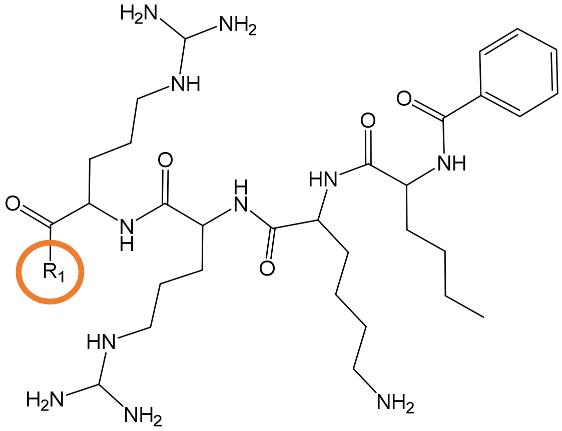	2<br>4<br>18<br>21	-OH<br>-NH2<br>-CF3<br>-B(OH)2	-	>500 µM<br>127.5 µM<br>0.85 µM<br>0.043 µM	[25]
CH3	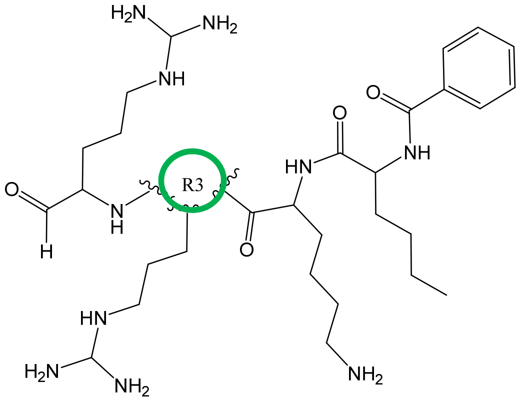	12<br>18<br>1	-Pro-<br>-DArg-<br>-Arg-	-	109 µM<br>115 µM<br>5.8 µM	[25]
CH7	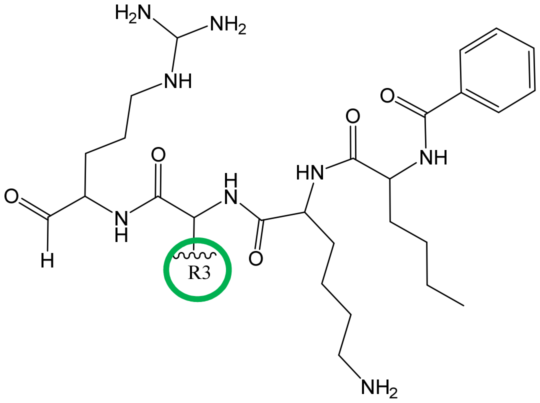	3<br>4<br>6<br>7	-Thr<br>-Arg<br>-Thr<br>-Arg	-	>500 µM<br>127.5 µM<br>>500 µM<br>5.8 µM	[25]

CV1	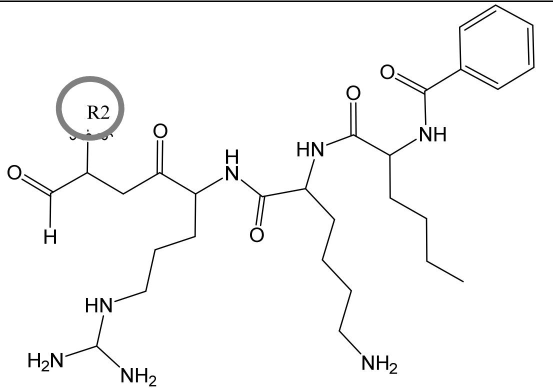	2<br>6	-Ala<br>-Phe	-	193 µM<br>15.9 µM	[25]
CV3	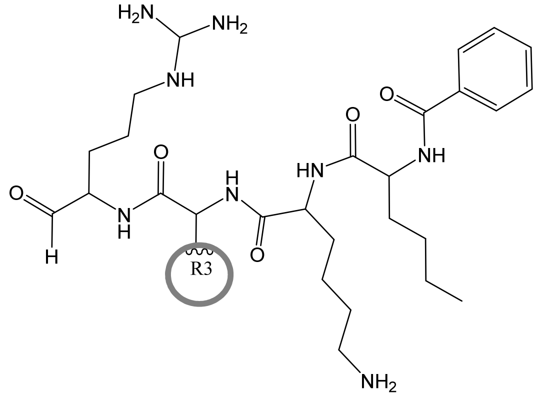	3<br>7	-Ala<br>-Phe	-	>500 µM<br>40.7 µM	[25]
DHV4	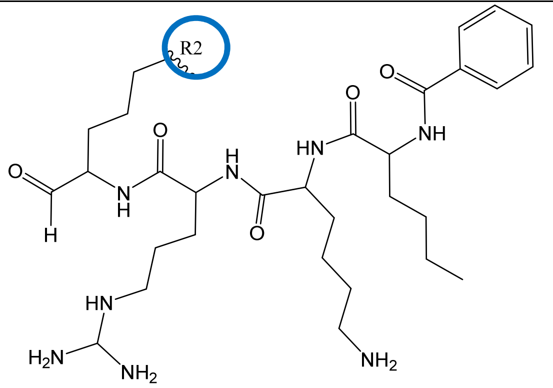	10<br>1<br>25<br>6<br>23	-Lys<br>-Arg<br>-(homo)Phe<br>-Phe<br>-(p-guanidinyl)Phe	-	20.5 µM<br>5.8 µM<br>>500 µM<br>15.9 µM<br>2.8 µM	[25]
DE2	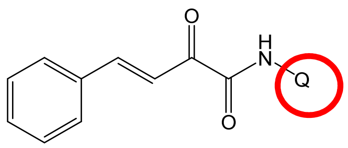	46<br>51<br>48	-C2H4(OCOCH3)<br>-CH(OH)(COOCH3)<br>-CH(CH3)(Phenyl)	35.8%<br>35.1%<br>0%	-	[26]
DV6	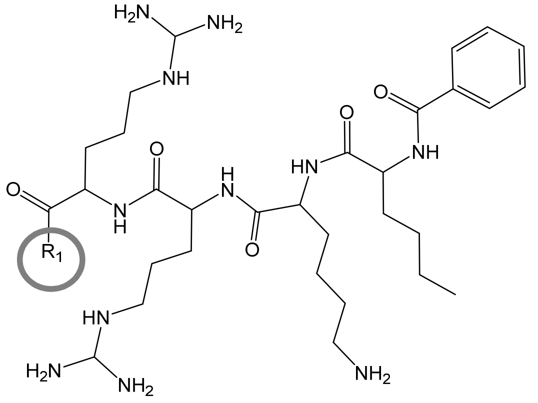	2<br>4<br>13a<br>13b	-OH<br>-NH2<br>-Benzoxazole<br>-Thiazole	-	>500 µM<br>127.5 µM<br>82.9 µM<br>42.8 µM	[25]

DV8	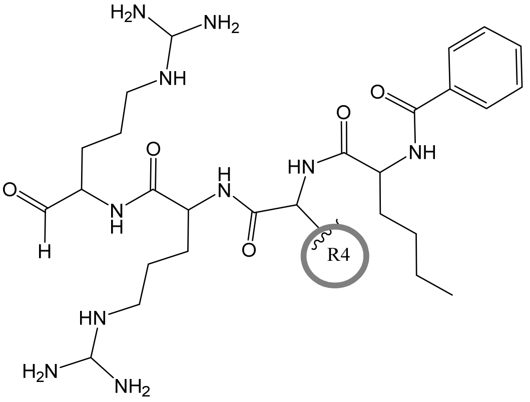	15<br>13<br>4<br>8	-NH(CH3)<br>-Pro<br>-Ala<br>-Phe	-	113.3 µM<br>61.4 µM<br>22.1 µM<br>15.8 µM	[25]

**(C) Anchor**	**Inhibitor scaffold**	**Compo-und**	**Moiety at anchor**	**IC_{50}**	**Ki**	**Ref.**
CEH1	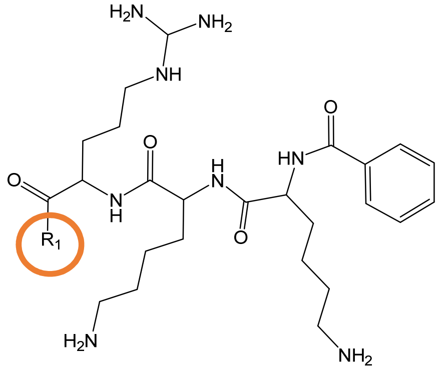	16<br>25	-H<br>-CHO	0.42 µM<br>0.271 µM	-	[27]
CH3	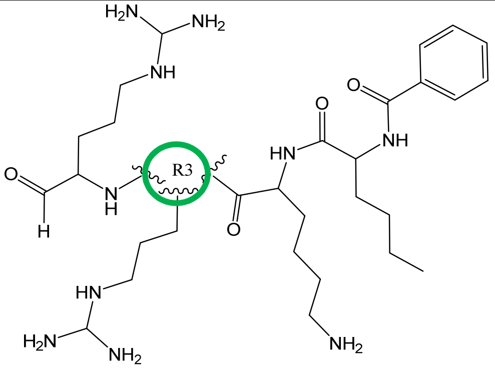	7<br>3<br>1	-DArg-<br>-N(CH3)-Arg-<br>-Arg-	128.6 mM<br>57.8 mM<br>4.1 mM	-	[28]
CH7	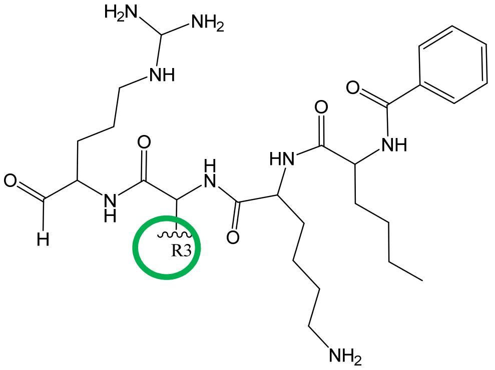	15<br>1<br>28	-Phe<br>-Arg<br>-Lys	108 mM<br>4.1 mM<br>1.9 mM	-	[28]

CV1	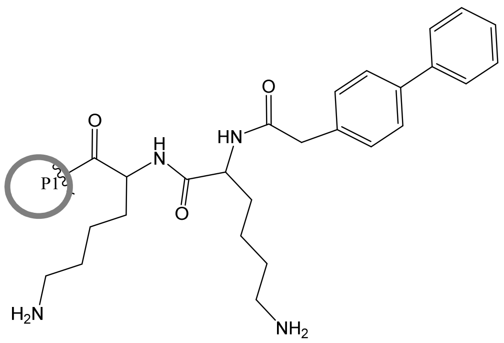	1<br>3<br>2<br>4<br>17	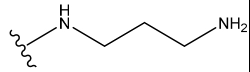<br>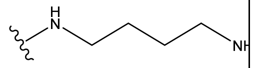<br>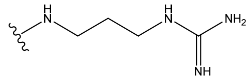<br>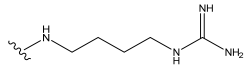<br>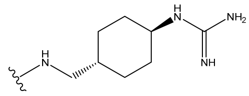	-	134 µM<br>31 µM<br>16 µM<br>3.9 µM<br>1.2 µM	[29]
CV3	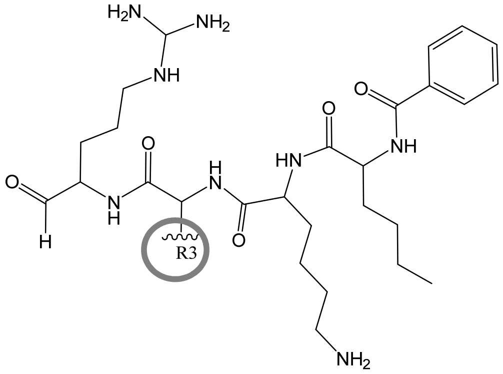	11<br>15	-Ala<br>-Phe	262 mM<br>108 mM	-	[28]
WHV4	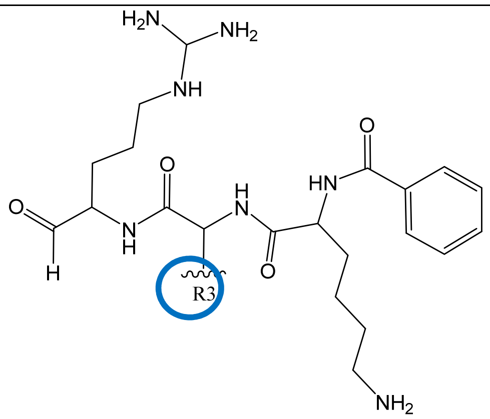	19<br>20<br>21<br>16<br>22	-Acetyl<br>-Benzoyl<br>-pAnisoyl<br>-H<br>-benzyl	112 µM<br>33 µM<br>8.3 µM<br>0.42 µM<br>24.2 µM	-	[27]
WHV8	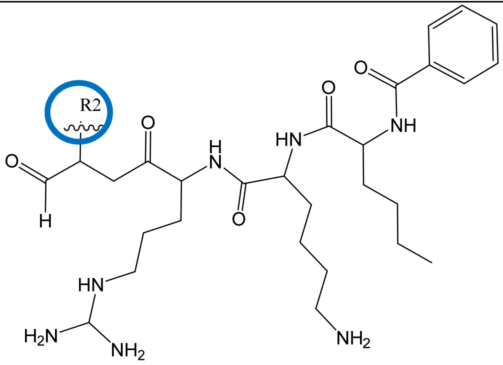	14<br>21<br>20<br>19<br>25	-Phe<br>-Tyr<br>-(pCN)Phe<br>-(pPh)Phe<br>-p(Guanidinyl)Phe	109.8 mM<br>41.5 mM<br>62 mM<br>22.7 mM<br>11.8 mM	-	[28]

WV5	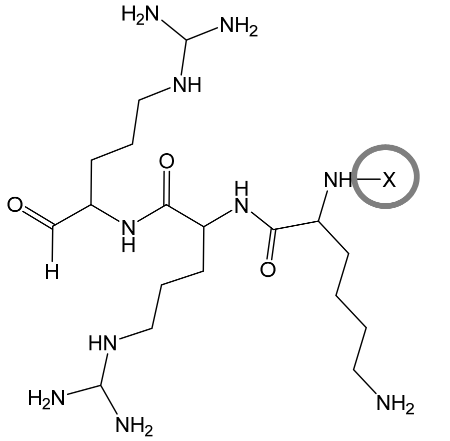	15<br>8<br>1<br>6<br>13<br>10<br>2	-(Phenyl)propionyl<br>-Propionyl<br>-nBenzoyl<br>-Acetyl<br>-2-Napthoyl<br>-4(Phenyl)phenylacetyl<br>-Phenylacetyl	19.7 µM<br>8.5 µM<br>2.6 µM<br>2.4 µM<br>1.8 µM<br>0.99 µM<br>0.39 µM	-	[27]
WV6	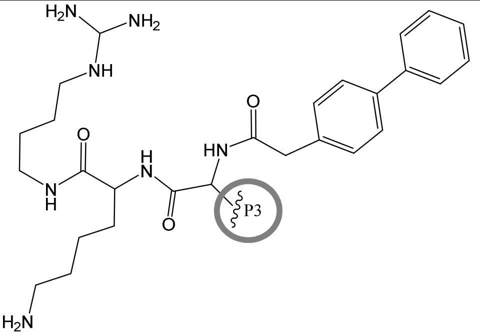	6<br>7<br>5<br>8	-Val<br>-Leu<br>-Ala<br>-Nle	>100 µM<br>>100 µM<br>53.7 µM<br>47.9 µM	-	[30]

References:

1. Noble CG, Seh CC, Chao AT, Shi PY: **Ligand-bound structures of the dengue virus protease reveal the active conformation**. *J Virol* 2012, **86**(1):438-446.
2. Erbel P, Schiering N, D'Arcy A, Renatus M, Kroemer M, Lim SP, Yin Z, Keller TH, Vasudevan SG, Hommel U: **Structural basis for the activation of flaviviral NS3 proteases from dengue and West Nile virus**. *Nat Struct Mol Biol* 2006, **13**(4):372-373.
3. Larkin MA, Blackshields G, Brown NP, Chenna R, McGettigan PA, McWilliam H, Valentin F, Wallace IM, Wilm A, Lopez R *et al*: **Clustal W and Clustal X version 2.0**. *Bioinformatics* 2007, **23**(21):2947-2948.
4. Shindyalov IN, Bourne PE: **Protein structure alignment by incremental combinatorial extension (CE) of the optimal path**. *Protein Eng* 1998, **11**(9):739-747.
5. Ashkenazy H, Erez E, Martz E, Pupko T, Ben-Tal N: **ConSurf 2010: calculating evolutionary conservation in sequence and structure of proteins and nucleic acids**. *Nucleic Acids Res* 2010, **38**(Web Server issue):W529-533.
6. Tong X, Chase R, Skelton A, Chen T, Wright-Minogue J, Malcolm BA: **Identification and analysis of fitness of resistance mutations against the HCV protease inhibitor SCH 503034**. *Antiviral Res* 2006, **70**(2):28-38.
7. Zeminian LB, Padovani JL, Corvino SM, Silva GF, Pardini MI, Grotto RM: **Variability and resistance mutations in the hepatitis C virus NS3 protease in patients not treated with protease inhibitors**. *Mem Inst Oswaldo Cruz* 2013, **108**(1):13-17.
8. Shoji I, Suzuki T, Chieda S, Sato M, Harada T, Chiba T, Matsuura Y, Miyamura T: **Proteolytic activity of NS3 serine proteinase of hepatitis C virus efficiently expressed in Escherichia coli**. *Hepatology* 1995, **22**(6):1648-1655.
9. Vallet S, Gouriou S, Nousbaum JB, Legrand-Quillien MC, Goudeau A, Picard B: **Genetic heterogeneity of the NS3 protease gene in hepatitis C virus genotype 1 from untreated infected patients**. *J Med Virol* 2005, **75**(4):528-537.
10. Beyer BM, Zhang R, Hong Z, Madison V, Malcolm BA: **Effect of naturally occurring active site mutations on hepatitis C virus NS3 protease specificity**. *Proteins* 2001, **43**(2):82-88.

11. Grakoui A, McCourt DW, Wychowski C, Feinstone SM, Rice CM: **Characterization of the hepatitis C virus-encoded serine proteinase: determination of proteinase-dependent polyprotein cleavage sites**. *J Virol* 1993, **67**(5):2832-2843.
12. Steinkuhler C, Biasiol G, Brunetti M, Urbani A, Koch U, Cortese R, Pessi A, De Francesco R: **Product inhibition of the hepatitis C virus NS3 protease**. *Biochemistry* 1998, **37**(25):8899-8905.
13. Lu L, Pilot-Matias TJ, Stewart KD, Randolph JT, Pithawalla R, He W, Huang PP, Klein LL, Mo H, Molla A: **Mutations conferring resistance to a potent hepatitis C virus serine protease inhibitor in vitro**. *Antimicrob Agents Chemother* 2004, **48**(6):2260-2266.
14. Trozzi C, Bartholomew L, Ceccacci A, Biasiol G, Pacini L, Altamura S, Narjes F, Muraglia E, Paonessa G, Koch U *et al*: **In vitro selection and characterization of hepatitis C virus serine protease variants resistant to an active-site peptide inhibitor**. *J Virol* 2003, **77**(6):3669-3679.
15. Valle RP, Falgout B: **Mutagenesis of the NS3 protease of dengue virus type 2**. *J Virol* 1998, **72**(1):624-632.
16. Salaemae W, Junaid M, Angsuthanasombat C, Katzenmeier G: **Structure-guided mutagenesis of active site residues in the dengue virus two-component protease NS2B-NS3**. *J Biomed Sci* 2010, **17**:68.
17. Jia F, Fan J, Zhang B, Yuan Z: **Mutagenesis of D80-82 and G83 residues in West Nile Virus NS2B: effects on NS2B-NS3 activity and viral replication**. *Virol Sin* 2013, **28**(1):16-23.
18. Chappell KJ, Stoermer MJ, Fairlie DP, Young PR: **Mutagenesis of the West Nile virus NS2B cofactor domain reveals two regions essential for protease activity**. *J Gen Virol* 2008, **89**(Pt 4):1010-1014.
19. Chappell KJ, Stoermer MJ, Fairlie DP, Young PR: **Insights to substrate binding and processing by West Nile Virus NS3 protease through combined modeling, protease mutagenesis, and kinetic studies**. *J Biol Chem* 2006, **281**(50):38448-38458.
20. Jiang Y, Andrews SW, Condroski KR, Buckman B, Serebryany V, Wenglowsky S, Kennedy AL, Madduru MR, Wang B, Lyon M *et al*: **Discovery of danoprevir (ITMN-191/R7227), a highly selective and potent inhibitor of hepatitis C virus (HCV) NS3/4A protease**. *J Med Chem* 2014, **57**(5):1753-1769.
21. Bogen SL, Ruan S, Liu R, Agrawal S, Pichardo J, Prongay A, Baroudy B, Saksena AK, Girijavallabhan V, Njoroge FG: **Depeptidization efforts on P3-P2' alpha-ketoamide inhibitors of HCV NS3-4A serine protease: effect on HCV replicon activity**. *Bioorg Med Chem Lett* 2006, **16**(6):1621-1627.
22. Prongay AJ, Guo Z, Yao N, Pichardo J, Fischmann T, Strickland C, Myers J, Jr., Weber PC, Beyer BM, Ingram R *et al*: **Discovery of the HCV NS3/4A protease inhibitor (1R,5S)-N-[3-amino-1-(cyclobutylmethyl)-2,3-dioxopropyl]-3- [2(S)-[[[(1,1-dimethylethyl)amino]carbonyl]amino]-3,3-dimethyl-1-oxobutyl]- 6,6-dimethyl-3-azabicyclo[3.1.0]hexan-2(S)-carboxamide (Sch 503034) II. Key steps in structure-based optimization**. *J Med Chem* 2007, **50**(10):2310-2318.
23. Venkatraman S, Bogen SL, Arasappan A, Bennett F, Chen K, Jao E, Liu YT, Lovey R, Hendrata S, Huang Y *et al*: **Discovery of (1R,5S)-N-[3-amino-1-(cyclobutylmethyl)-2,3-dioxopropyl]- 3-[2(S)-[[[(1,1-dimethylethyl)amino]carbonyl]amino]-3,3-dimethyl-1-oxobutyl]- 6,6-dimethyl-3-azabicyclo[3.1.0]hexan-2(S)-carboxamide (SCH 503034), a selective, potent, orally bioavailable hepatitis C virus NS3 protease inhibitor: a potential therapeutic agent for the treatment of hepatitis C infection**. *J Med Chem* 2006, **49**(20):6074-6086.
24. Scola PM, Wang AX, Good AC, Sun LQ, Combrink KD, Campbell JA, Chen J, Tu Y, Sin N, Venables BL *et al*: **Discovery and early clinical evaluation of BMS-605339, a potent and orally efficacious tripeptidic acylsulfonamide NS3 protease inhibitor for the treatment of hepatitis C virus infection**. *J Med Chem* 2014, **57**(5):1708-1729.
25. Yin Z, Patel SJ, Wang WL, Chan WL, Ranga Rao KR, Wang G, Ngew X, Patel V, Beer D, Knox JE *et al*: **Peptide inhibitors of dengue virus NS3 protease. Part 2: SAR study of tetrapeptide aldehyde inhibitors**. *Bioorg Med Chem Lett* 2006, **16**(1):40-43.
26. Steuer C, Gege C, Fischl W, Heinonen KH, Bartenschlager R, Klein CD: **Synthesis and biological evaluation of alpha-ketoamides as inhibitors of the Dengue virus protease with antiviral activity in cell-culture**. *Bioorg Med Chem* 2011, **19**(13):4067-4074.
27. Schuller A, Yin Z, Brian Chia CS, Doan DN, Kim HK, Shang L, Loh TP, Hill J, Vasudevan SG: **Tripeptide inhibitors of dengue and West Nile virus NS2B-NS3 protease**. *Antiviral Res* 2011, **92**(1):96-101.
28. Knox JE, Ma NL, Yin Z, Patel SJ, Wang WL, Chan WL, Ranga Rao KR, Wang G, Ngew X, Patel V *et al*: **Peptide inhibitors of West Nile NS3 protease: SAR study of tetrapeptide aldehyde inhibitors**. *J Med Chem* 2006, **49**(22):6585-6590.
29. Hammamy MZ, Haase C, Hammami M, Hilgenfeld R, Steinmetzer T: **Development and characterization of new peptidomimetic inhibitors of the West Nile virus NS2B-NS3 protease**. *ChemMedChem* 2013, **8**(2):231-241.
30. Lim HA, Ang MJ, Joy J, Poulsen A, Wu W, Ching SC, Hill J, Chia CS: **Novel agmatine dipeptide inhibitors against the West Nile virus NS2B/NS3 protease: a P3 and N-cap optimization study**. *Eur J Med Chem* 2013, **62**:199-205.
